# PLFYNet-based edge-deployable detection system for Ginkgo biloba leaf diseases

**DOI:** 10.3389/fpls.2025.1679455

**Published:** 2025-11-27

**Authors:** Jun Wang, Siyuan Gu, Maocheng Zhao

**Affiliations:** 1 College of Information Science and Technology& Artificial Intelligence, Nanjing Forestry University, Nanjing, China; 2 College of Mechanical and Electronic Engineering, Nanjing Forestry University, Nanjing, China

**Keywords:** leaf-used Ginkgo biloba, lightweight, disease detection, attention mechanism, LAMP

## Abstract

**Introduction:**

Target detection is a pivotal technology for precise monitoring of leaf-used Ginkgo biloba diseases in precision agriculture. However, complex plantation environments impose significant constraints on existing detection systems, manifesting as degraded detection accuracy, suboptimal efficiency, and prohibitive computational overhead for edge deployment. This study aims to develop a lightweight deep learning model tailored for real-time disease detection on resource-constrained embedded devices.

**Methods:**

First, a comprehensive multi-class dataset was constructed, containing 7,158 augmented images covering three disease categories: chlorosis, insect pest, and physical damage. Five lightweight architectures were systematically evaluated, and an optimized reconstructed backbone network was adopted. To maintain architectural efficiency, attention mechanisms, an improved detection head, and efficient convolution techniques were integrated, along with a custom feature fusion module designed to address small target feature loss—forming the base model LCNet-FusionYOLO. Subsequently, Layer-Adaptive Magnitude-based Pruning (LAMP) was applied to reduce model scale while enhancing performance, yielding the final PLFYNet model.

**Results:**

The PLFYNet model achieves 94.5% mAP@0.5 with only 3.0M parameters, surpassing the baseline YOLOv7-tiny by 4.8% while using merely half the parameters. Deployment on the Jetson Orin Nano embedded platform demonstrates real-time inference at 50.5 FPS, validating its practical applicability in field scenarios.

**Discussion:**

This work establishes a paradigm for developing high-precision, computationally efficient disease detection systems. By balancing accuracy and resource efficiency, PLFYNet provides a practical edge-based monitoring solution for sustainable Ginkgo biloba cultivation, addressing the key deployment challenges of existing detection systems in complex agricultural environments.

## 1 Introduction

Ginkgo biloba is a multifunctional agricultural commodity in China, serving ornamental, culinary, and pharmaceutical purposes—its medicinal value lies primarily in foliage containing flavonoids and terpenoid lactones. These bioactive compounds benefit cardiovascular health and cerebrovascular circulation, promote brain cell metabolism (Wang et al., 2022; Zhang et al., 2024), and exhibit antioxidant, anti-inflammatory, and other physiological benefits (Zhang et al., 2023a). This pharmaceutical leaf value makes foliar diseases (e.g., anthracnose caused by Colletotrichum fructicola) particularly damaging, as infections reduce both yield and medicinal compound content.

As the global leader in Ginkgo biloba cultivation, China’s extensive subtropical plantations have positioned it as a crucial research center for advancing leaf disease management strategies (Sun et al., 2017). The intensification of global climate change and ecological disruptions has amplified disease pressures on leaf-harvested Ginkgo biloba (Zhang et al., 2023b). Insect infestations frequently induce leaf chlorosis and premature defoliation, significantly impeding plant development. Under conducive environmental conditions—particularly elevated temperature and humidity—pests such as Scirtothrips can proliferate rapidly throughout plantations, resulting in substantial economic losses (Mirab-balou et al., 2012). Furthermore, various stressors including natural disasters (hail, frost, excessive precipitation) and anthropogenic factors (mechanical injury, pesticide misapplication) continue to threaten sustainable Ginkgo biloba production.

Recent advances in deep learning, particularly the integration of convolutional neural networks (CNNs) and target detection algorithms, have revolutionized automated plant disease detection by enhancing accuracy and efficiency.They enable early identification of infections.This reduces manual monitoring efforts.It also supports timely pest control measures (Gkountakos et al., 2024; Jiang et al., 2020). However, Ginkgo biloba’s unique characteristics—medicinally valuable foliage with fine-textured lesions and dense plantation canopies—create unmet needs that generic crop detection models fail to address.

Girshick et al.’s R-CNN established the foundational CNN-based detection framework (Girshick et al., 2014), but Ren et al. later noted that even the optimized Faster R-CNN retains excessive computational complexity (Ren et al., 2017)—a critical limitation for edge deployment in remote Ginkgo plantations where high-performance servers are unavailable. The YOLO series addressed real-time demands, yet Omer et al.’s lightweight YOLOv5l, optimized for cucumber diseases, demonstrated that crop-specific customization is essential (Omer et al., 2024); their model achieved high accuracy for cucumber lesions but lacked adaptability to Ginkgo’s tiny, irregular spots (e.g., early anthracnose lesions under 2mm). Even YOLOv7-tiny, a common edge-focused variant, struggles with Ginkgo-specific challenges: its unidirectional PANet fusion leads to high false-negative rates in dense canopies, and conventional CIoU loss fails to precisely localize morphologically diverse lesion. Compounding these issues, Gkountakos et al.’s review highlighted that existing models lose up to 15% accuracy in variable field lightin—a prevalent condition in Ginkgo plantations.

Although these vision-based algorithms enable rapid identification and localization of both emerging and established infections, facilitating timely targeted interventions such as precision fungicide applications, ultimately mitigating economic losses (Wang et al., 2025a; Zhu et al., 2023), several limitations still exist. To address these limitations, we propose PLFYNet, an enhanced lightweight architecture based on YOLOv7-tiny. This model targets the identified gaps through four key innovations. First, it integrates Bottleneck Transformer to capture long-range lesion dependencies (Xie et al., 2025a). This resolves the canopy occlusion issue highlighted. Second, it uses BiECAFusion for bidirectional cross-scale feature interaction. This fixes the small lesion loss problem in crop-specific model. Third, it adopts Shape-IoU loss for accurate bounding box optimization. This overcomes the localization inaccuracies of YOLOv7-tiny noted by Zhao et al. (2023). Fourth, it incorporates DyHead (Duan et al., 2024) to enable robust target representation under variable lighting. We selected the reconstructed PP-LCNet as the backbone after evaluating five lightweight architectures: MobileNetV3, GhostNet, ShuffleNetV2, PP-PicoDet, and PP-LCNet. This backbone significantly reduces computational complexity while maintaining detection performance. Additionally, the model integrates the Efficient Channel Attention (ECA) mechanism. This enhances feature discrimination between similar lesions, such as insect-induced chlorosis and abiotic yellowing. Comprehensive evaluations confirm that LCNET-FusionYOLO is superior to both the original and conventional models in terms of mAP@0.5, false-positive, and false-negative metrics. Further optimization through LAMP pruning, which involves adaptive layer-wise redundancy elimination, results in the final PLFYNet architecture. This architecture has reduced parameters (3.0M), improved real-time capability (50.5 FPS on Jetson Orin Nano), and accelerated inference. It directly addresses the deployment barrier identified in Ren et al. (2017) Faster R-CNN analysis and enables high-precision field detection in dense canopy environments.

The research methodology encompasses three primary phases:

1. Dataset Development: We acquired 3, 600 high-resolution Ginkgo leaf images and applied comprehensive augmentation strategies to expand the dataset, effectively mitigating overfitting while enhancing model robustness. Precise disease annotation was performed using Labelme software.
2. Architectural Innovation: We developed an optimized detection framework based on YOLOv7-tiny, reconstructing the backbone with PP-LCNet’s DepthSepConv modules to minimize computational overhead. Systematic evaluation of five lightweight architectures (MobileNetV3, GhostNet, ShuffleNetV2, PP-PicoDet, and PP-LCNet) identified the optimal configuration. The selected Light-YOLO variant underwent comparative analysis with SE, CBAM, and ECA attention mechanisms. Performance enhancement was achieved through four integrated components: Bottleneck Transformer for global context modeling, BiECAFusion for multi-scale feature fusion, Shape-IoU for bounding box refinement, and DyHead for target representation. LAMP pruning provided final model compression. Evaluation metrics encompassed parameter count, precision, recall, mAP@0.5, and memory footprint.
3. Edge Deployment: The optimized model was successfully deployed on the Jetson Orin Nano embedded platform, achieving 50.5 FPS real-time detection while maintaining accuracy. Comparative analysis validated superior inference speed and precision relative to baseline models.

## 2 Materials and methods

### 2.1 Preparation of the dataset

#### 2.1.1 Source of experimental dataset

**Figure 1:**
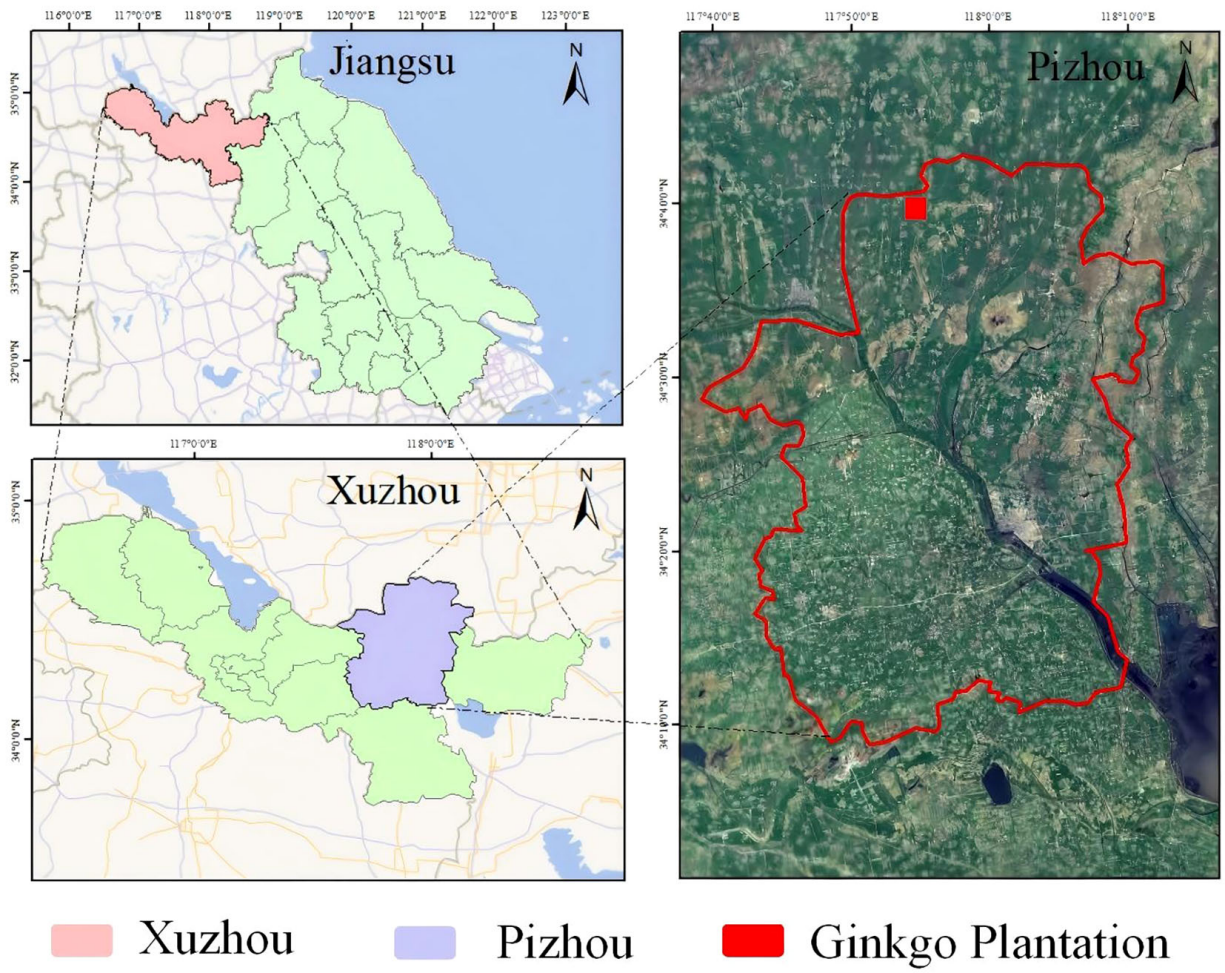
Main dataset collection sites.

Data collection was conducted at two strategic locations: the primary site at Changrong Agricultural Development Co., Ltd. in Pizhou, Jiangsu Province (117°52’E, 34°37’N)—China’s premier “Ginkgo Hometown” hosting the nation’s largest commercial plantations—and supplementary sampling from Nanjing Forestry University. The principal research facility features 12 cultivation zones covering 117.07 hectares of leaf-oriented Ginkgo production, with spatial distribution presented in **Figure 1**.

#### 2.1.2 Design of datasets

In this work, classifications were developed based on field research. We tracked the one-year growth cycle of Pizhou City’s ginkgo plantations, and the dataset covers representative disease symptoms across annual growth cycles, with main diseases distinguished by professional plantation disease managers. June-September is pest outbreak season; high temperatures bring tender shoots, and pests like Scirtothrips (short cycles, strong drug resistance) outbreak massively. They gnaw leaves rapidly, cutting plantation yields, and these pests are hard to detect (hide in leaves, have protective coloration) while gnawed leaves show obvious symptoms. Thus, such cases are classified as “insect_pest” by leaf characteristics. Physical damage (hailstorms, rain/snow, mechanical operations) has distinct features, so it is categorized as “physical_damage”. Chlorosis, a biotic stress disease, stems from fungi (Alternaria spp., etc.), yellowing is typical of such fungal infections on ginkgo leaves, and it is fully classified as “chlorosis” based on field research. A simplified labeling scheme was adopted: 0-chlorosis (symptomatic manifestation), 1-insect pest, 2-physical damage.

To strengthen model generalization capabilities and prevent overfitting, extensive data augmentation protocols were applied. Transformations included photometric variations (brightness, hue), contrast manipulations, and spatial rescaling, yielding an enriched dataset of 7, 158 images. Dataset partitioning followed an 8:1:1 distribution for training, validation, and testing cohorts, respectively, ensuring methodological rigor in model evaluation. we have conducted generalization verification experiments using the public dataset PlantDoc. PlantDoc is an open-source public dataset for plant disease detection, containing 2, 598 manually annotated images covering 13 plant species and 17 diseases, with an 85% training set and 15% validation set split (Singh et al., 2020).

**Figure 2:**
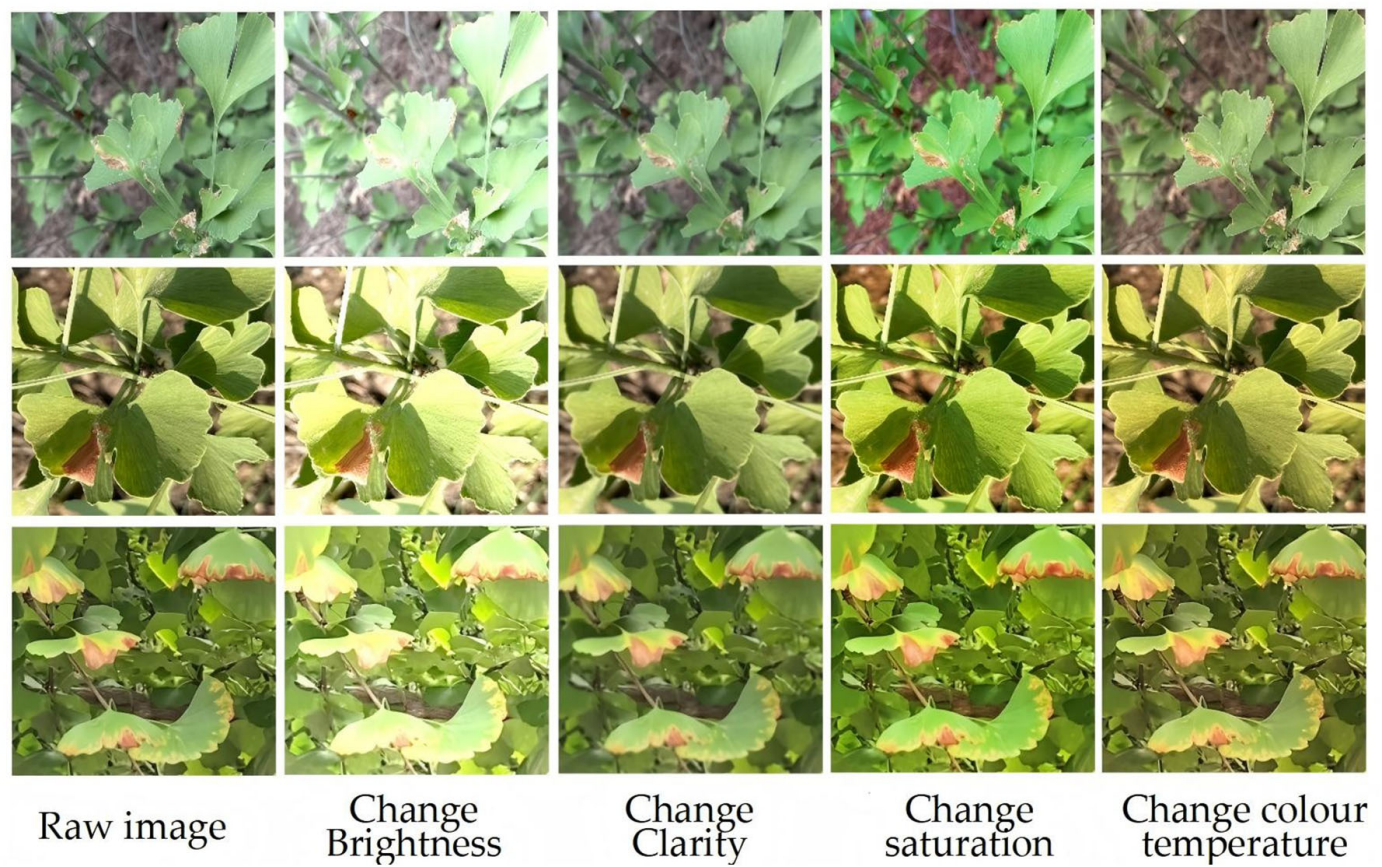
Enhanced image of the dataset.

LabelMe software facilitated precise annotation of the augmented dataset, generating 9, 582 labeled instances categorized as: chlorosis (n=1, 779), insect pest (n=5, 260), and physical damage (n=2, 543). Visual representation of these categories appears in **Figure 2**, arranged hierarchically with insect-induced damage, physical trauma, and chlorosis symptoms displayed in successive rows. Class balancing was achieved through targeted augmentation of underrepresented categories, particularly enhancing insect pest samples to ensure equitable distribution.

**Figure 3:**
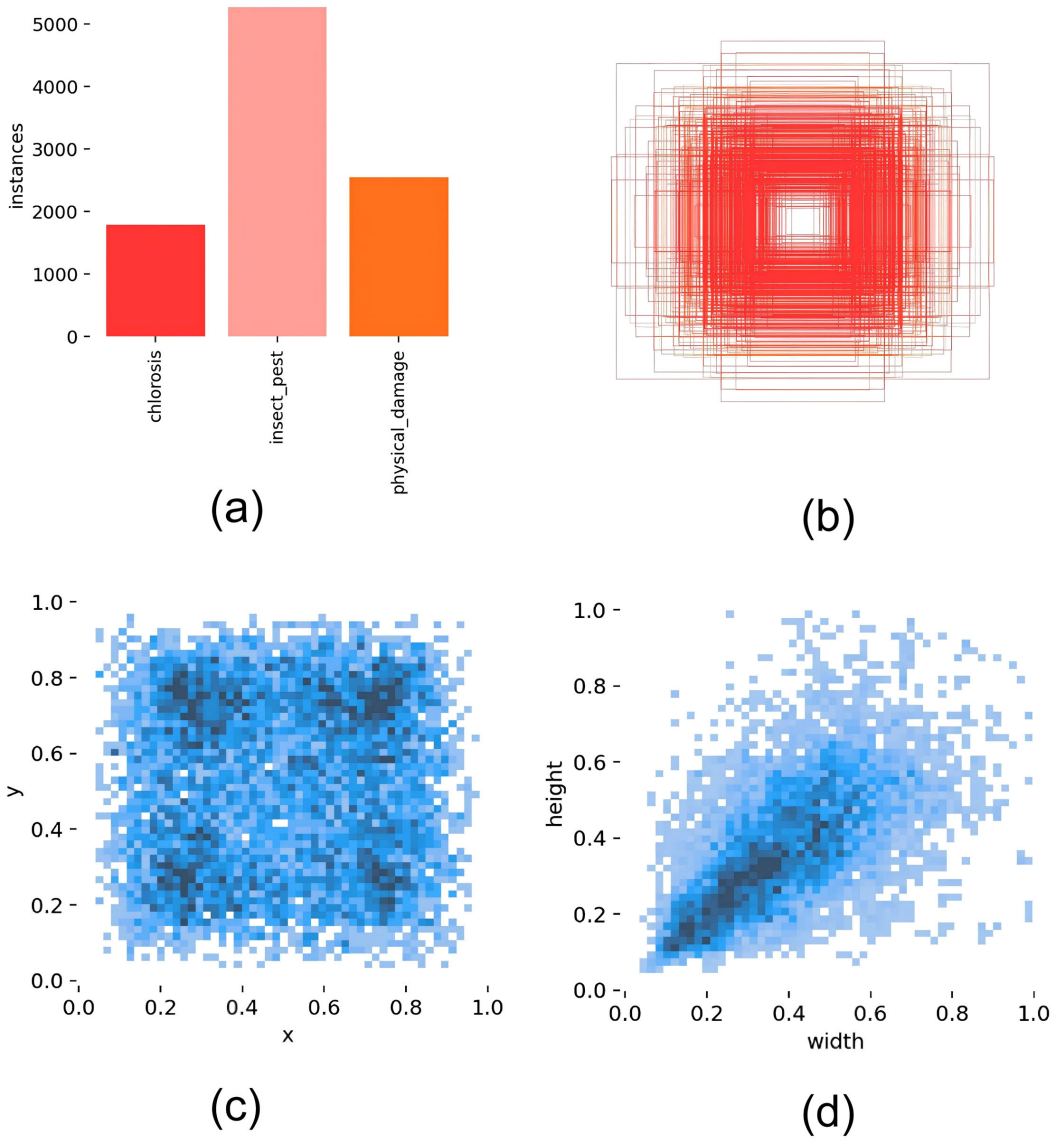
Dataset characteristics analysis: **(a)** Class distribution of disease labels; **(b)** Bounding box size distribution via center point aggregation; **(c)** Spatial distribution of label centroid coordinates; **(d)** Width-height distribution of bounding boxes for disease regions.

**Figure 3** a comprehensive analysis of dataset properties. (a) Class distribution of disease labels :The class distribution histogram displays three categories—chlorosis (red), insect pest (pink), and physical damage (orange)—with insect pest exhibiting the highest frequency due to morphological complexity, necessitating extensive training samples. Loss function weighting compensates for class imbalance. (b) Bounding box size distribution via center point aggregation :Concentrically arranged rectangles illustrate bounding box size distributions centered at coordinate origins. The radial expansion pattern demonstrates damage scale progression from minor peripheral lesions to substantial central deterioration, with intermediate sizes showing highest prevalence. (c) Spatial distribution of label centroid coordinates :Scatter plot analysis of normalized centroid coordinates (range: 0-1) reveals uniform spatial distribution across leaf surfaces, extending from periphery (x, y approaching 0 or 1) to center (x, y ≈ 0.5), confirming comprehensive spatial representation without clustering artifacts. (d) Width-height distribution of bounding boxes for disease regions :Width-height correlation analysis exhibits strong diagonal alignment, indicating predominantly isometric damage patterns (characteristic of puncture wounds), while off-diagonal dispersal represents anisotropic lesions (typical of linear abrasions). The pronounced diagonal concentration confirms regular geometric damage patterns in Ginkgo biloba pathology.

### 2.2 YOLOv7 object detection model

The YOLOv7-tiny architecture offers an optimal balance between model compactness and detection speed, rendering it particularly well-suited for embedded applications on Jetson Orin Nano hardware, enabling real-time pathological assessment of Ginkgo biloba foliage (Zhao et al., 2023; Wang et al., 2023).

The YOLOv7-tiny architecture is structured into four hierarchical stages: initial input processing for image preparation, a feature extraction backbone, an intermediate neck for multi-scale aggregation, and a final detection module. The backbone employs a combination of CBL units (convolutional blocks with batch normalization and LeakyReLU activation) and ELAN modules, which leverage multi-path architectures for enhanced feature representation, complemented by MP layers for progressive spatial reduction. Feature fusion occurs through SPPCSPC modules (Xu et al., 2024), which integrate Spatial Pyramid Pooling with Cross-Stage Partial connections, effectively expanding receptive fields while maintaining parameter efficiency across multiple scales.

**Figure 4:**
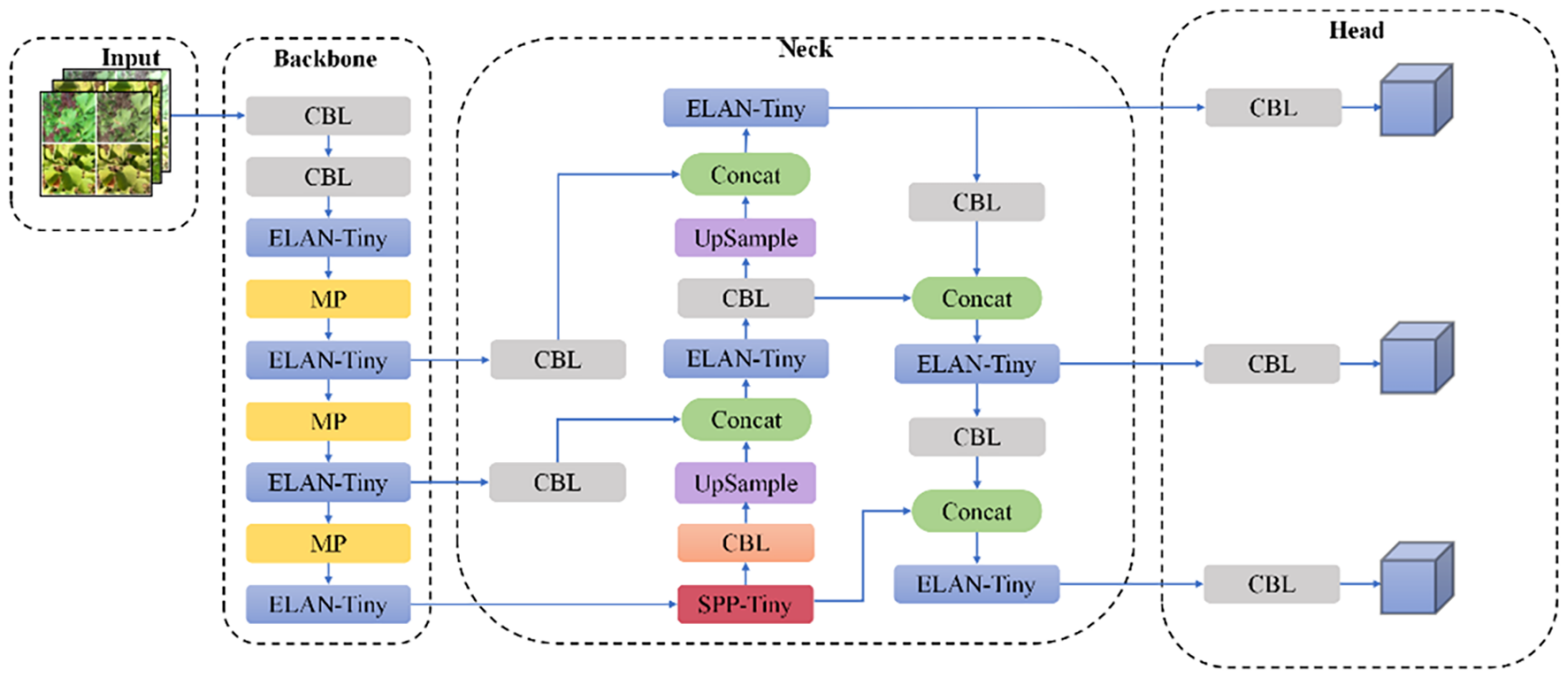
YOLOv7 network structure diagram.

The SPPCSPC module employs a bifurcated processing strategy for input features. One pathway directly processes features using a CBL block, while the alternate pathway implements a more complex structure: after initial CBL transformation, it diverges into four parallel streams comprising an identity mapping and three max-pooling operations with distinct kernel dimensions. This dual-pathway design achieves optimal balance between computational efficiency and detection performance via multi-level feature aggregation, as depicted in **Figure 4**, showing the complete YOLOv7 architecture.

### 2.3 PLFYNet object detection model

#### 2.3.1 Network reconstruction and optimization

A systematic evaluation of five lightweight architectures—MobileNetV3, GhostNet, ShuffleNetV2, PP-PicoDet, and PP-LCNet—was conducted to identify the optimal backbone replacement for YOLOv7-tiny in Ginkgo disease detection applications (Wang et al., 2025b; Shen et al., 2023; Han and Yang, 2021; Lu et al., 2025a). Through comprehensive benchmarking, PP-LCNet emerged as the superior choice, demonstrating exceptional performance metrics.

As a CNN architecture specifically engineered for mobile deployment (Xue and Wang, 2025), PP-LCNet employs a streamlined design featuring sequential convolutional and pooling operations tailored for real-time inference. The architecture’s foundation consists of DepthSepConv blocks, which utilize dual convolutional layers paired with H-Swish activation functions for computational efficiency (Huang et al., 2023). Strategic integration of Squeeze-and-Excitation mechanisms in select modules yields DepthSepConvSE variants with enhanced representational capacity.

**Figure 5:**
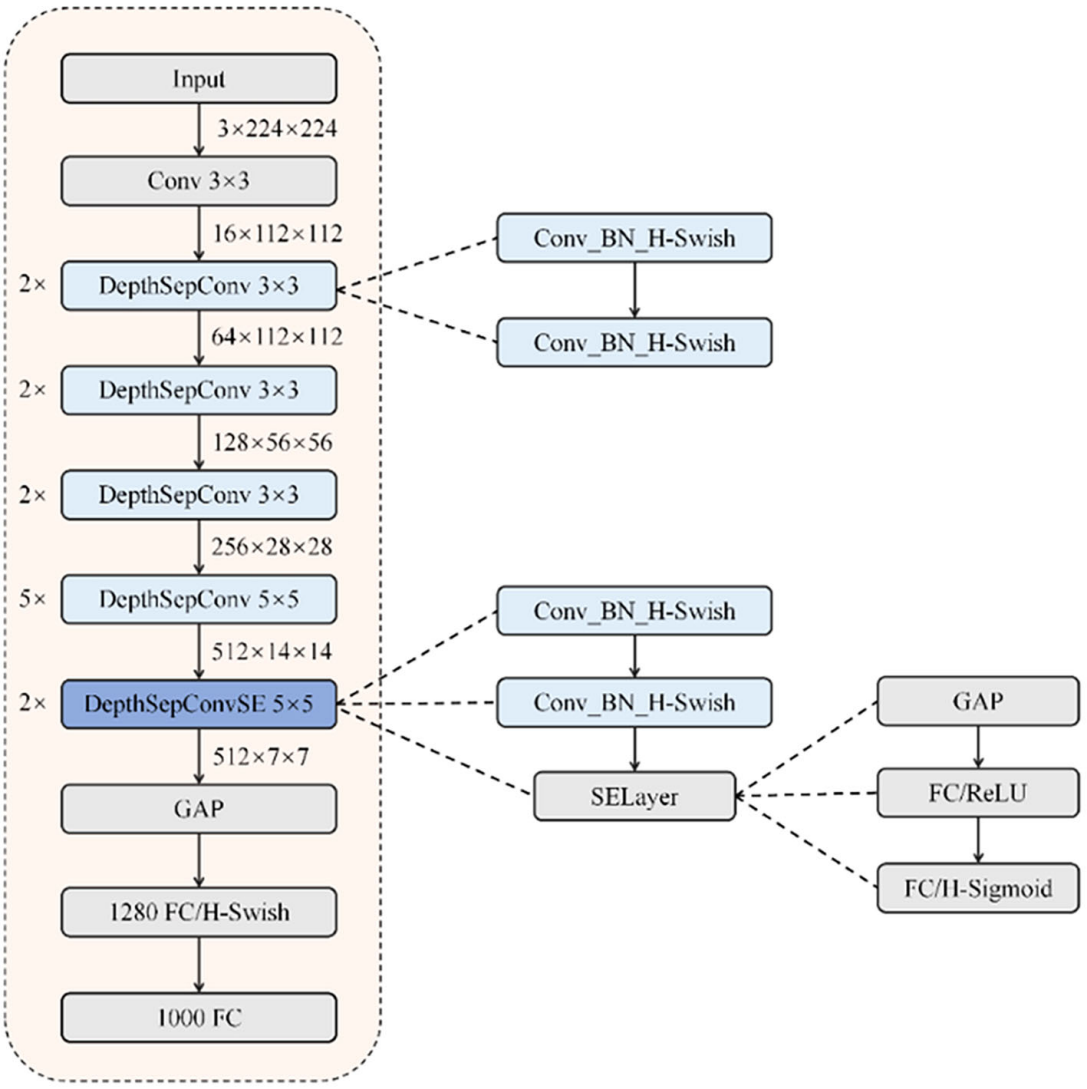
PP-LCNet network structure.

A distinctive feature of PP-LCNet is its architectural departure from standard classification models through the incorporation of a 1280-channel 1×1 convolutional layer following global average pooling. This design innovation delivers improved classification performance without compromising inference speed. The overall structure of PP-LCNet is shown in in **Figure 5**.

**Figure 6:**
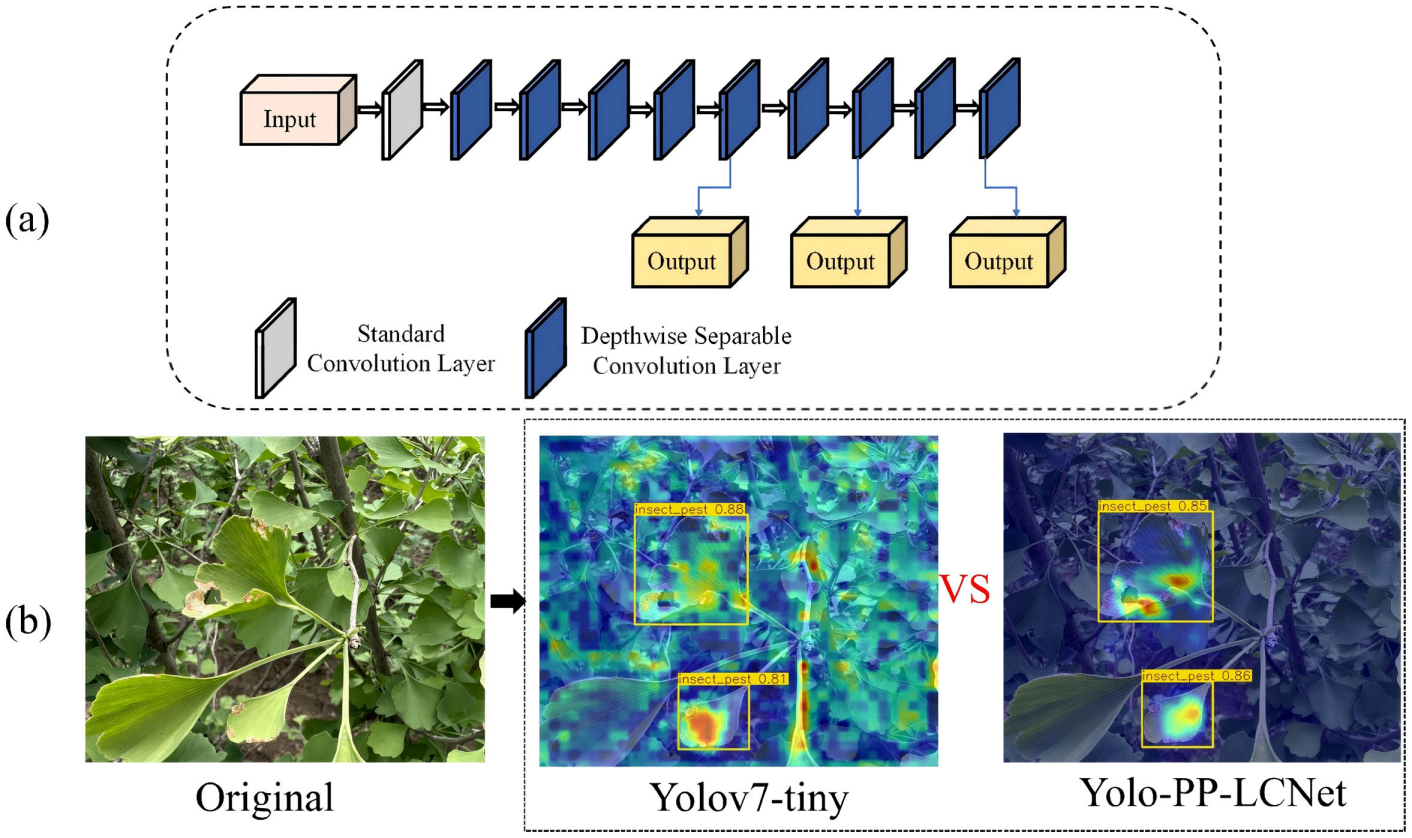
Architecture of backbone module and its detection performance visualization: **(a)** The structure of the backbone network with the DepthSepConv basic module; **(b)** Detection output results.

**Figure 6** depicts the architectural diagram of the modified YOLOv7-tiny incorporating a PP-LCNet-inspired backbone reconstruction based on DepthSepConv modules. The redesigned backbone initiates with a conventional convolutional layer, subsequently progressing through a cascade of DepthSepConv blocks featuring dual kernel configurations: 3×3 and 5×5 convolutions. This heterogeneous kernel design facilitates multi-scale feature extraction by capturing information across diverse receptive field dimensions.

**Table 1** delineates the architectural specifications and parametric configurations of the modified backbone network through five essential components: (i) repetition factor (n), indicating the iteration count for each module; (ii) parameter volume (Params), quantifying trainable weights per stage; (iii) module designation, specifying the computational blocks utilized; (iv) configuration details, encompassing comprehensive parametric settings; and (v) output dimensionality, representing spatial resolution and channel depth (H × W × C).

**Table 1:** Backbone network structure and parameterization.

Serial number	n	Params	Module	Configuration	Output size
0	1	464	Conv	[3, 16, 3, 2, 1]	16×320×320
1	1	752	DepthSepConv	[16, 32, 3, 1]	32×320×320
2	1	2528	DepthSepConv	[32, 64, 3, 2]	64×160×160
3	1	4928	DepthSepConv	[64, 64, 3, 1]	64×160×6
4	1	9152	DepthSepConv	[64, 128, 3, 2]	128×80×80
5	1	18048	DepthSepConv	[128, 128, 3, 1]	128×80×80
6	1	34688	DepthSepConv	[128, 256, 3, 2]	256×40×40
7	5	364800	DepthSepConv	[256, 256, 5, 1]	256×40×40
8	1	139008	DepthSepConv	[256, 512, 5, 2]	512×20×20
9	1	276992	DepthSepConv	[512, 512, 5, 1]	512×20×20

Parametric distinctions between modules are evident: Standard Convolution operates with a five-tuple specification (input channels, output channels, kernel dimensions, stride, padding), whereas DepthSepConv employs a reduced four-parameter scheme (input/output channels, kernel size, stride), notably omitting padding specifications and SE attention integration.

#### 2.3.2 The light-YOLO network model integrating the attention mechanism

A comprehensive benchmarking study was conducted on the Ginkgo biloba pathology dataset to assess three prominent attention mechanisms—SE, CBAM, and ECA (Ai et al., 2025; Fareed et al., 2025; Waghumbare et al., 2024)—within the Light-YOLO framework under controlled experimental protocols. Performance quantification employed a four-metric evaluation suite comprising parameter efficiency, detection precision, recall rate, and mAP0.5 Empirical results demonstrated ECA’s dominant performance across all assessed dimensions, warranting its selection for the proposed architecture.

#### 2.3.3 Neck network improvement

The YOLOv7-tiny architecture prioritizes computational efficiency through reduced network depth and parameter count, facilitating deployment on resource-constrained platforms. This architectural parsimony, however, compromises detection performance, manifesting as elevated false negative rates in complex visual environments. Addressing these limitations, we introduce three targeted architectural modifications tailored for Ginkgo biloba pathology detection, emphasizing enhanced discrimination of diminutive lesions within visually cluttered canopy environments:

1. Bottleneck Transformer (BoT) Module: Incorporates self-attention mechanisms to model long-range spatial dependencies, augmenting global context awareness. This integration substantially enhances detection sensitivity and localization accuracy for small-scale pathological features.
2. Bidirectional ECA-enhanced Feature Fusion (BiECAFusion): Implements bidirectional feature propagation across multiple scales while preserving fine-grained spatial details. This dual-pathway architecture synergistically combines high-level semantic representations with low-level textural features, yielding superior detection robustness.
3. Shape-IoU Loss Formulation: Supersedes conventional CIoU with a geometry-aware loss function (Zhao et al., 2024), achieving improved alignment between predicted and ground-truth bounding boxes. This refinement particularly benefits the localization of morphologically diverse disease manifestations.

**Figure 7:**
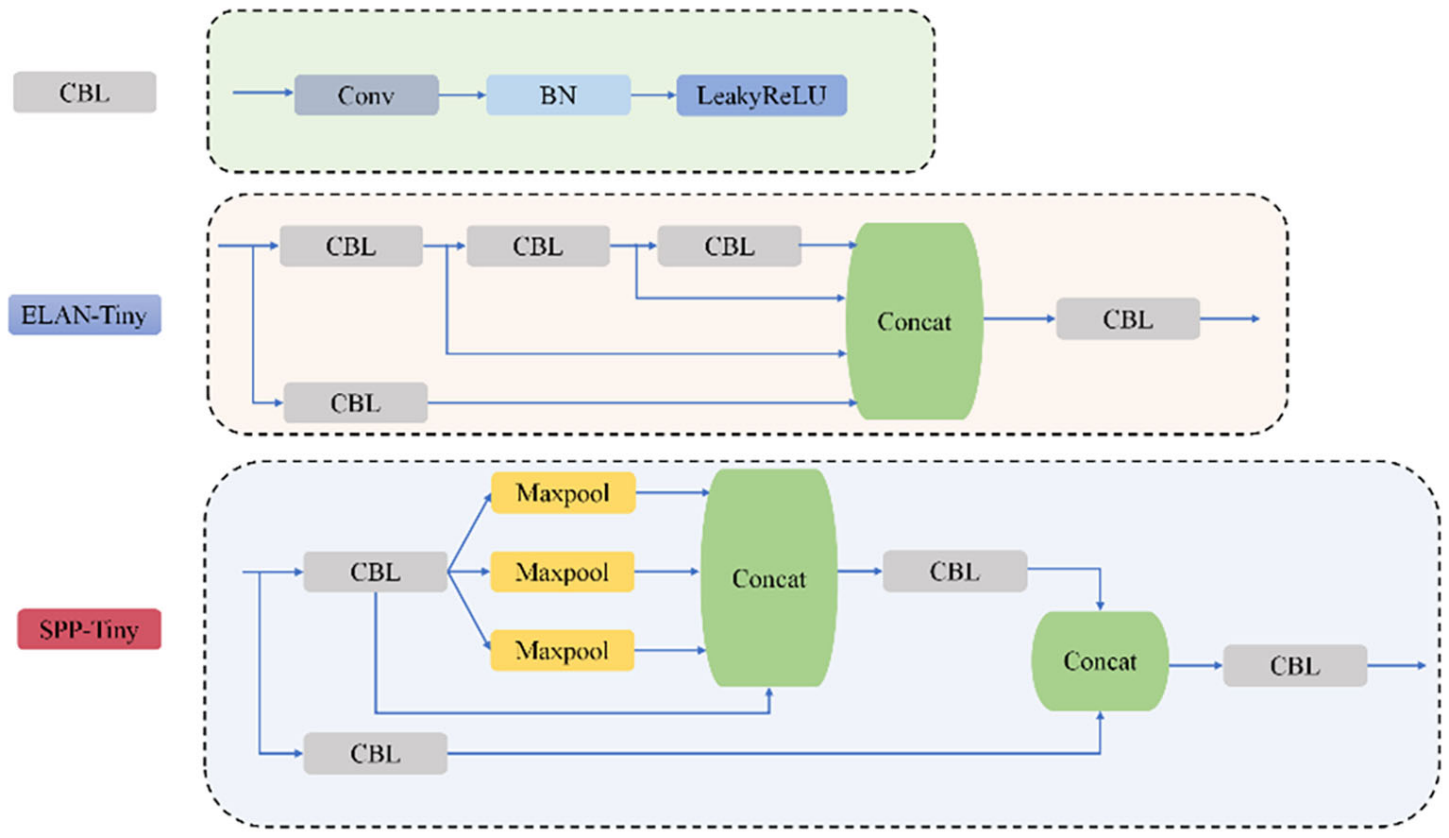
CBL, ELAN, and SPPCPC modules.

The structure of the CBL, ELAN, and SPPCPC modules is shown in **Figure 7**.

#### 2.3.4 Fused bottleneck transformer module

**Figure 8:**
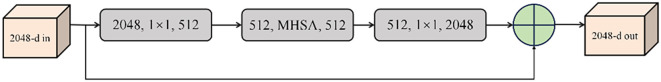
Bottleneck transformer module.

The Bottleneck Transformer architecture substitutes ResNet’s conventional 3×3 convolutions with a hybrid attention-convolution design (Srinivas et al., 2021). The module architecture consists of two principal components: a multi-head self-attention (MHSA) mechanism and a nonlinear projection block. The MHSA component performs dimensional decomposition of input features, enabling parallelized computation while establishing global spatial dependencies (Lu et al., 2025b). Following attention computation, the nonlinear projection block employs dual linear transformations to introduce essential nonlinearities. Hierarchical stacking of these modules, as illustrated in **Figure 8**, yields a deep architecture that synergistically leverages convolutional and transformer paradigms (Yang et al., 2025; Yu and Zhou, 2023). This design logic aligned with the discriminative feature modeling philosophy in DFPNet, which enhances fine-grained target representation via structured feature pyramid (Xie et al., 2024). This design philosophy achieves enhanced feature representation through global context modeling while maintaining computational efficiency. Within the YOLOv7 framework, this integration yields simultaneous improvements in detection accuracy and computational performance, facilitating real-time inference with reduced deployment overhead.

#### 2.3.5 Fused BiECAFusion structure

**Figure 9:**
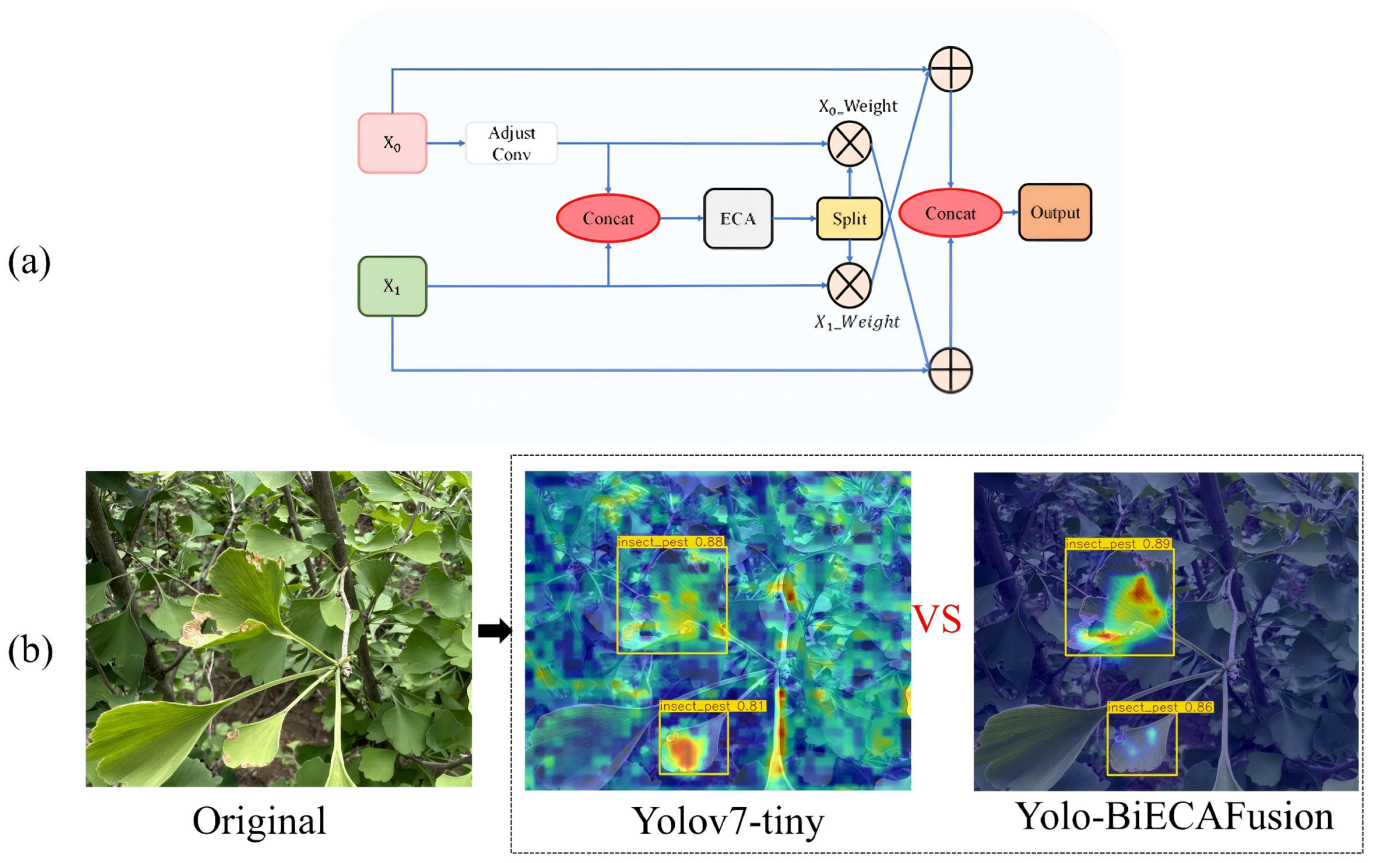
Architecture of BiECAFusion module and its detection performance visualization: **(a)** Bidirectional ECA-enhanced feature fusion structure; **(b)** Detection output results.

The original YOLOv7 architecture employs PANet (Path Aggregation Network) for feature aggregation through unidirectional pathways (Zhang et al., 2023c). This approach exhibits inherent limitations when applied to Ginkgo biloba pathology detection, particularly in preserving discriminative feature representations across scales, resulting in suboptimal learning dynamics. To mitigate these deficiencies, we propose BiECAFusion (Bidirectional ECA-enhanced Feature Fusion), a novel feature aggregation module that facilitates bidirectional information flow while incorporating channel-wise attention mechanisms. The architectural design is detailed in **Figure 9**.

BiECAFusion is a fundamental redesign of feature fusion strategies. It is specifically developed to tackle the challenges of small lesion detection in complex foliar environments. The module operates in three stages. First, 1×1 convolutions are used for channel dimensionality harmonization. This ensures compatible feature representations across different hierarchical levels. Second, the Efficient Channel Attention (ECA) mechanism is integrated. It dynamically recalibrates channel responses, highlighting discriminative features and suppressing background noise. This is crucial for detecting tiny pathological indicators with minimal spatial coverage. Finally, the bidirectional fusion paradigm retains both fine-grained spatial details and high-level semantic information. It achieves this through reciprocal feature enhancement (Tang et al., 2025; Xie et al., 2025b).

The BiECAFusion Module differs significantly from the traditional concat fusion in FPN/PAN in terms of fusion logic and attention mechanism. Traditional FPN/PAN achieves fusion by simply concatenating high-level (semantic-rich, low-resolution) and low-level (detail-rich, high-resolution) features, where all feature channels are treated equally without adaptive weighting. BiECAFusion replaces the original SE attention with ECA attention and adopts a dual-branch interaction mechanism: it first adjusts the channel dimensions of input features via 1×1 convolution to ensure compatibility, concatenates the features, applies ECA attention to generate channel-wise weights, splits the weights into branches corresponding to input features, and then fuses each branch feature with the weighted feature of the other branch (e.g., x0 + x1_weight, x1 + x0_weight), realizing bidirectional mutual enhancement of cross-level features. Besides, traditional concat lacks an attention mechanism and tends to retain redundant or irrelevant features (e.g., mixing non-lesion leaf textures with critical lesion edges), while BiECAFusion’s ECA attention can adaptively highlight effective channels, focus on high-frequency features like lesion edges, and suppress noise such as irrelevant leaf veins, solving the “feature confusion” problem in traditional fusion.

In this study, BiECAFusion demonstrates three key advantages. First, it enhances lesion edge discrimination: lesion edges are crucial for detecting ginkgo leaf diseases (e.g., yellow spots, brown blight) but easily confused with dense leaf veins, and its ECA attention prioritizes high-frequency edge features, while dual-branch interaction strengthens the correlation between semantic (lesion category) and geometric (edge shape) information, facilitating small lesion recognition. Second, it enables efficient cross-level feature interaction: unlike the one-way fusion of FPN/PAN (high-to-low or low-to-high), BiECAFusion allows x0 to benefit from x1’s details and x1 from x0’s semantics, adapting to ginkgo scenarios where lesions vary greatly in size (from tiny spots to large patches) and require balanced use of multi-scale features. Third, it has a lightweight design: ECA attention removes fully connected layers, which aligns with the study’s goal of developing edge-deployable ginkgo disease detection models, ensuring fusion efficiency without increasing computational burden.

- Dimensional Harmonization: Employing 1×1 convolutions for channel normalization facilitates seamless cross-scale integration while minimizing computational complexity.
- Adaptive Channel Recalibration: The ECA mechanism generates channel-specific attention weights with minimal overhead, selectively amplifying features relevant to small-scale pathological patterns.
- Bidirectional Feature Synthesis: The reciprocal enhancement strategy (x_0_ + w_1_x_1_, x_1_ + w_0_x_0_) establishes complementary interactions between spatially-rich shallow features and semantically-rich deep representations.

#### 2.3.6 Loss function improvement

The YOLOv7 architecture uses a multi-component loss. It covers confidence, localization and classification objectives. For bounding box regression, the baseline uses Complete Intersection over Union (CIoU). CIoU combines overlap ratio, centroid distance and aspect ratio consistency. But CIoU has flaws. It performs poorly when predicted and ground truth aspect ratios are similar. This causes suboptimal convergence.

We adopt Shape-IoU for ginkgo leaf disease detection. It explicitly models geometric links between predicted and target boxes. For irregular ginkgo lesions (e.g., leaf spots), it optimizes shape-aware localization. This fixes CIoU’s flaws and boosts localization accuracy for ginkgo scenarios.

The Shape-IoU loss function is calculated as follows (Equations 1-6):

(1) $$IoU=\frac{\left|B\cap B^{gt}\right|}{\left|B\cup B^{gt}\right|}$$

(2) $$ww=\frac{2\times {\left(w^{gt}\right)}^{scale}}{{\left(w^{gt}\right)}^{scale}+{\left(h^{gt}\right)}^{scale}}$$

(3) $$hh=\frac{2\times {\left(h^{gt}\right)}^{scale}}{{\left(w^{gt}\right)}^{scale}+{\left(h^{gt}\right)}^{scale}}$$

(4) $$distance^{shape}=hh\times {\left(x_{c}-x_{c}^{gt}\right)}^{2}/c^{2}+ww\times {\left(y_{c}-y_{c}^{gt}\right)}^{2}/c^{2}$$

(5) $${\text{Ω}}^{shape}=\sum_{t=w,h}{\left(1-e^{-\omega_{t}}\right)}^{\theta },\theta =4$$

(6) $$\left\{ \begin{array}{c} \omega_{w}=hh\times \frac{\left|w-w^{gt}\right|}{\text{max}(w,w^{gt})}\\ \omega_{h}=ww\times \frac{\left|h-h^{gt}\right|}{\text{max}(h,h^{gt})} \end{array}\right.$$

The scale parameter is empirically calibrated based on the object size distribution within the training corpus. Directional weight coefficients, denoted as ww and hh for horizontal and vertical axes respectively, are dynamically computed from the geometric properties of ground truth annotations. The comprehensive Shape-IoU loss integrates IoU, shape distance, and shape penalty, as shown in Equation 7.

(7) $$L_{Shape-IoU}=1-IoU+distance^{shape}+0.5\times {\text{Ω}}^{Shape}$$

#### 2.3.7 Detection head improvement

DyHead constitutes an innovative detection head architecture that employs multi-dimensional self-attention to enhance feature discrimination across scale, spatial, and semantic dimensions (Dai et al., 2021). The framework’s distinctive characteristic lies in its unified attention mechanism that operates orthogonally across feature pyramid levels (L), spatial locations (S), and channel dimensions (C), facilitating holistic feature refinement. Through this tripartite attention strategy, DyHead augments the detection head’s representational power without imposing computational penalties, thereby achieving an optimal balance between detection performance and computational efficiency (Gong et al., 2024). Formally, for a feature tensor F ∈ R^(L×S×C), the self-attention mechanism is expressed as in Equation 8:

(8) W(F)=π(F)·F

In this formulation, π(·) represents the attention transformation function. Although fully-connected architectures could theoretically model such high-dimensional interactions, the computational complexity of simultaneously learning across all tensor dimensions renders this approach intractable. Consequently, we adopt a factorized attention strategy, decomposing the operation into three consecutive transformations, with each targeting a specific dimensional axis independently.

(9) $$\text{W}(F)={\text{π}}_{\text{C}}({\text{π}}_{\text{S}}({\text{π}}_{\text{L}}(F)\cdot F)\cdot F)\cdot F$$

**Figure 10:**
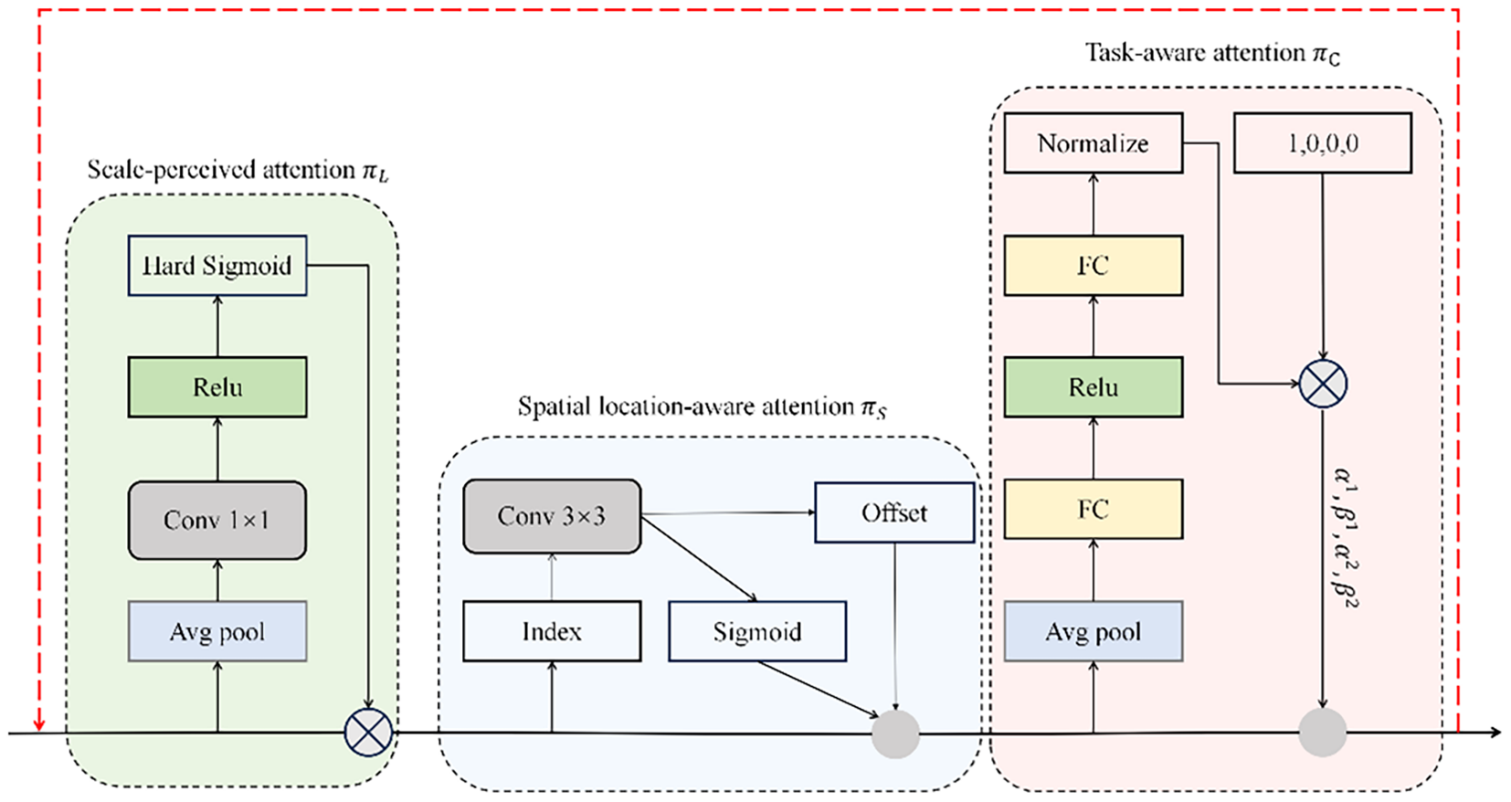
DyHead structure.

${\text{π}}_{\text{L}}(\cdot )$, ${\text{π}}_{\text{S}}(\cdot )$ and ${\text{π}}_{\text{C}}(\cdot )$ represent dimension-specific attention transformations corresponding to level, spatial, and channel axes, respectively. Sequential execution of these operators ensures computational tractability while preserving inter-dimensional dependencies. The formulation in Equation 9 inherently supports recursive composition, facilitating the construction of deep architectures through cascaded ${\text{π}}_{\text{L}}$, ${\text{π}}_{\text{S}}$, and ${\text{π}}_{\text{C}}$ modules, as depicted in the architectural diagram of **Figure 10**.

#### 2.3.8 Improved network structure

**Figure 11:**
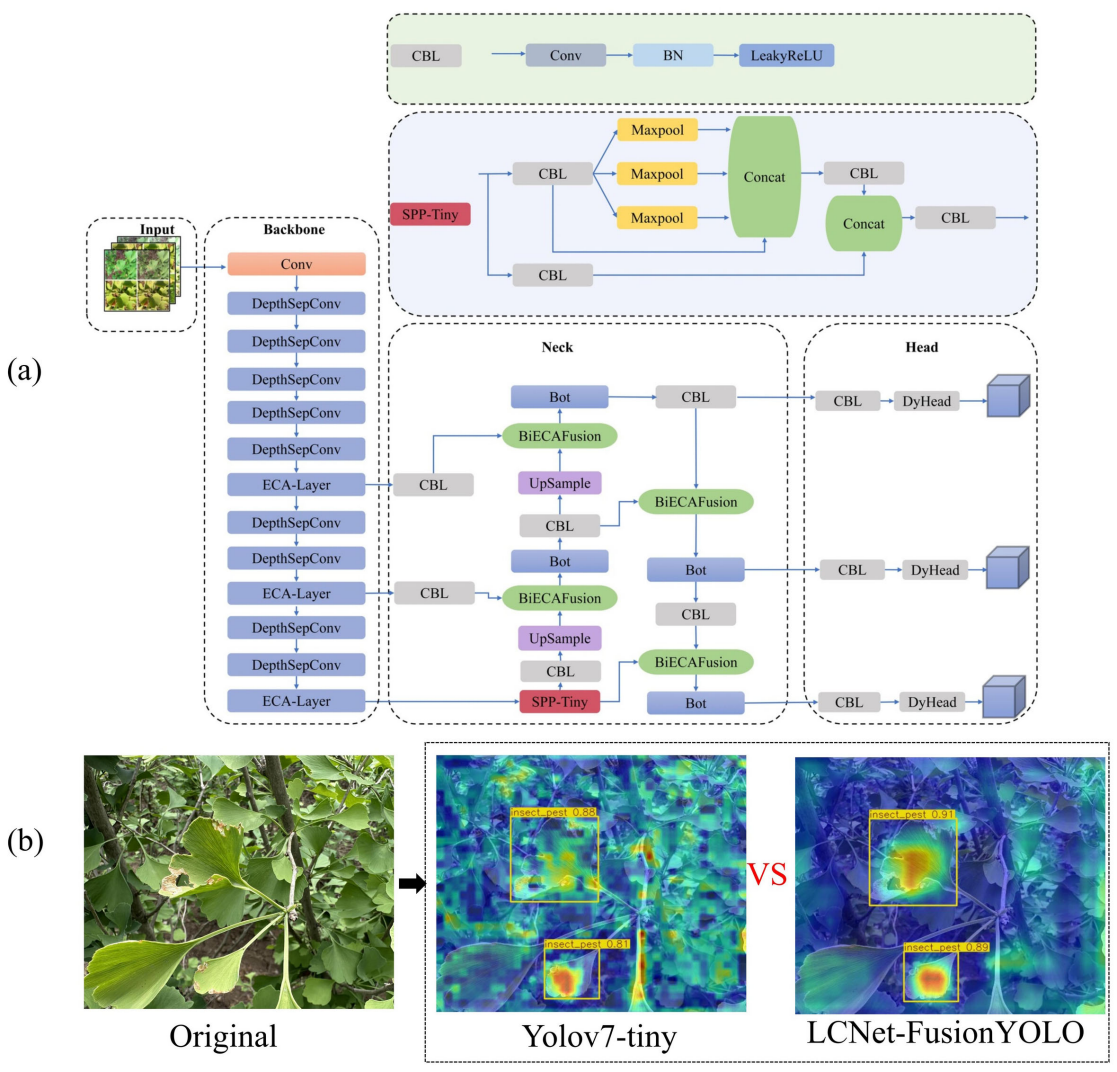
Architecture of LCNET-FusionYOLO model and its detection performance visualization: **(a)** LCNET-FusionYOLO Network Architecture and **(b)** Detection output results.

The culmination of these architectural innovations yields LCNET-FusionYOLO, whose comprehensive topology is delineated in **Figure 11**.

#### 2.3.9 LAMP model pruning

Following model convergence, we implement LAMP (Layer-Adaptive Magnitude-based Pruning) to further compress the architecture for edge deployment (Lee et al., 2020). High-resolution disease image computational demands present significant constraints for resource-limited embedded platforms. To address these challenges, LAMP uses adaptive layer-wise sparsification, where connection importance is quantified by normalized weight magnitudes. The algorithm computes relative significance scores by normalizing squared weight values against the aggregate magnitude of retained parameters within each layer, enabling automatic derivation of layer-specific pruning ratios. This approach ensures optimal model compression while maintaining detection fidelity for embedded applications (Yuan et al., 2025).

The methodology fundamentally balances sparsity optimization with performance preservation through adaptive global pruning. Weight salience is determined via magnitude-based scoring coupled with ℓ_2_ distortion minimization at the network level. The algorithmic pipeline comprises:

Sorting according to the magnitude of weights: Parameters within each network layer are arranged in descending order based on their absolute values, establishing a magnitude-based hierarchy. LAMP Score Calculation: The algorithm computes normalized importance metrics by evaluating the squared magnitude of each weight relative to the layer’s weight distribution, yielding calibrated significance scores. Global Pooling and Pruning: Layer-specific scores undergo aggregation into a unified importance repository, followed by comprehensive ranking and systematic parameter elimination based on global thresholds. This methodology achieves optimal compression while preserving essential model capabilities.

### 2.4 Evaluation indicators

Model selection criteria encompass five comprehensive evaluation metrics: detection precision (P), sensitivity/recall (R), mean average precision (mAP@0.5), computational burden quantified through GFLOPS, and temporal efficiency measured via frames per second (FPS). The mAP@0.5 metric specifically denotes the averaged precision values computed across all disease categories at an intersection-over-union threshold of 0.5.

(10) $$\text{P}=\frac{\text{TP}}{\text{TP+FP}}$$

(11) $$\text{R}=\frac{\text{TP}}{\text{TP+FN}}\;$$

(12) $$\text{AP}=\int_{0}^{1}\text{P}(\text{R})\text{dR}$$

(13) $$\text{mAP}=\frac{\sum_{\text{k}=1}^{\text{N}}{\text{AP}}_{\text{k}}}{\text{N}}$$

The core evaluation metrics for model performance—Precision, Recall, AP, and mAP—are calculated using Equations 10–13. Within these formulations, TP represents true positive detections where diseased instances are correctly identified, FP indicates false positives arising from misclassification of healthy samples as diseased, FN denotes false negatives occurring when pathological cases are erroneously classified as healthy, and N signifies the total number of disease categories under consideration.

### 2.5 Experimental environment

The algorithmic model experiments in this paper were conducted on the Ubuntu operating system, with a CPU of 12 vCPU Intel(R) Xeon(R) Platinum 8352V CPU @ 2.10GHz, and GPUs of NVIDIA RTX 3080x2 (20GB). The Python programming language was adopted, and the PyTorch 3.8, CUDA 11.8, and CUDNN deep learning framework was used for model training and inference. The main parameter settings shown in **Table 2**. All baseline models (YOLOv7-tiny, SSD, Faster R-CNN, etc.) were trained under the same conditions. and the dataset comprised 7, 158 images collected in this study.

**Table 2:** Main parameter settings.

Parameter	Values
Initial learning rate	0.01
Weight decay	0.0005
Epoch	300

## 3 Results

### 3.1 Comparison of ablation experiments

#### 3.1.1 Analysis of network model lightweighting results

**Table 3** presents a comprehensive performance analysis of YOLOv7-tiny variants reconstructed with five alternative backbone architectures: MobileNetV3, GhostNet, ShuffleNetV2, PP-PicoDet, and PP-LCNet. The comparative evaluation encompasses computational metrics (parameter count and memory footprint) alongside detection performance indicators (precision, recall, and mAP@0.5).

**Table 3:** Lightweighting results for different backbone network models.

Basic model	MobileNetV3	GhostNet	ShuffleNetV2	PP-PicoDet	PP-LCNet	Parameter (million)	Memory(MB)	Precision (%)	Recall(%)	mAP@0.5(%)
YOLOv7-tiny						6.0	12.4	89.9	83.7	89.7
	✓					1.5	3.2	88.6	83.5	89.9
		✓				7.2	14.1	90.6	87.6	91.9
			✓			4.3	8.5	91.1	87.2	91.1
				✓		4.3	8.7	82.6	84.7	87.7
					✓	4.4	8.6	91.4	87.7	91.2

Experimental analysis reveals a consistent trade-off between parameter efficiency and detection performance across all evaluated architectures. While MobileNetV3 achieves substantial parameter reduction (74.5%), its marginal mAP@0.5 gain (0.2%) coupled with notable precision degradation (1.3%) renders it suboptimal for this application. GhostNet exhibits counterproductive behavior, expanding parameters by 56% relative to baseline, thus eliminating it from consideration. The remaining architectures—ShuffleNetV2, PP-PicoDet, and PP-LCNet—demonstrate comparable parameter compression (approximately 28%), with PP-LCNet emerging as the superior variant, achieving 91.2% mAP@0.5. This configuration yields a 26.8% parameter reduction while enhancing recall by 4.0% and preserving precision levels, substantiating the architectural choice.

#### 3.1.2 Analysis of light-YOLO network models incorporating attention mechanisms

Building upon the Light-YOLO architecture, we conducted systematic ablation studies incorporating three prominent attention mechanisms—SE, CBAM, and ECA—to assess their respective contributions to detection performance. **Table 4** presents a comprehensive comparative analysis of these attention-augmented variants.

**Table 4:** Modelling results incorporating different attention mechanisms.

Basic model	PP-LCNet	SE	CBAM	ECA	Parameter(million)	Precision (%)	Recall (%)	mAP@0.5 (%)
YOLOv7-tiny	✓				4.4	91.4	87.7	91.2
	✓	✓			4.8	91.3	87.6	91.4
	✓		✓		4.5	87.5	89.3	90.6
	✓			✓	4.4	88.8	88.8	93.1

Empirical analysis reveals distinct performance characteristics across the evaluated attention mechanisms. SE module integration incurs an 8.6% parameter overhead while yielding marginal performance shifts—a 0.1% precision reduction offset by equivalent recall improvement and 0.2% mAP@0.5 gain. CBAM demonstrates inferior performance, with mAP@0.5 falling 0.6% below SE-augmented models, suggesting suboptimal feature recalibration for this application. Conversely, ECA exhibits superior efficiency, maintaining parameter parity with the baseline while achieving substantial performance gains: 1.1% recall enhancement and 1.9% mAP@0.5 improvement. These results establish ECA as the optimal attention mechanism for Ginkgo pathology detection, warranting its integration into the PP-LCNet backbone for enhanced detection fidelity.

#### 3.1.3 Performance comparison of fusion improvement modules

Following the empirical validation of ECA as the optimal attention mechanism for the PP-LCNet backbone, we conducted systematic ablation studies to assess individual and combined contributions of the proposed enhancements. The evaluation encompassed baseline YOLOv7-tiny alongside variants incorporating BoT, BiECAFusion, Shape-IoU, and DyHead modifications, culminating in the fully integrated BBSD-YOLO architecture. Performance quantification employed a comprehensive metric suite—parameter count, memory footprint, precision, recall, and mAP@0.5—with comparative results tabulated in **Table 5**.

**Table 5:** Performance comparison of fusion improvement modules.

Basic model	BoT	BiECAFusion	Shape-IoU	DyHead	PP-LCNet	ECA	Parameter(million)	Memory(MB)	Precision (%)	Recall (%)	mAP@0.5 (%)
YOLOv7-tiny							6.0	12.4	89.9	83.7	89.7
	✓						8.9	17.2	92.5	90.7	93.1
		✓					6.0	8.6	89.4	86.3	90.1
			✓				6.0	11.6	92.4	91.6	93.2
				✓			5.9	10.5	91.2	90.8	91.2
	✓	✓	✓	✓			7.8	12.7	92.8	90.2	93.5
	✓	✓	✓	✓	✓	✓	6.2	10.2	94.1	91.8	94.1

**Table 5** demonstrates consistent performance improvements across all proposed architectural modifications relative to baseline YOLOv7-tiny. The BoT module, while increasing parameters by 48.2% (4.8MB memory overhead), yields substantial gains: 2.6% precision, 7.0% recall, and 3.4% mAP@0.5. BiECAFusion achieves parameter-efficient enhancement, maintaining computational complexity while delivering incremental improvements (0.48% precision, 2.6% recall, 0.4% mAP@0.5). Shape-IoU optimization significantly enhances localization accuracy, contributing 2.5% precision, 7.9% recall, and 3.5% mAP@0.5 gains. DyHead simultaneously reduces parameters while improving detection metrics (1.3% precision, 7.1% recall, 1.5% mAP@0.5). Synergistic integration of these components in BBSD-YOLO achieves optimal performance: 92.8% precision, 90.2% recall, and 93.5% mAP@0.5—representing a 4.9 percentage point improvement over baseline. The complete LCNET-FusionYOLO architecture further elevates performance to 94.1% precision, 91.8% recall, and 94.1% mAP@0.5.

**Figure 12:**
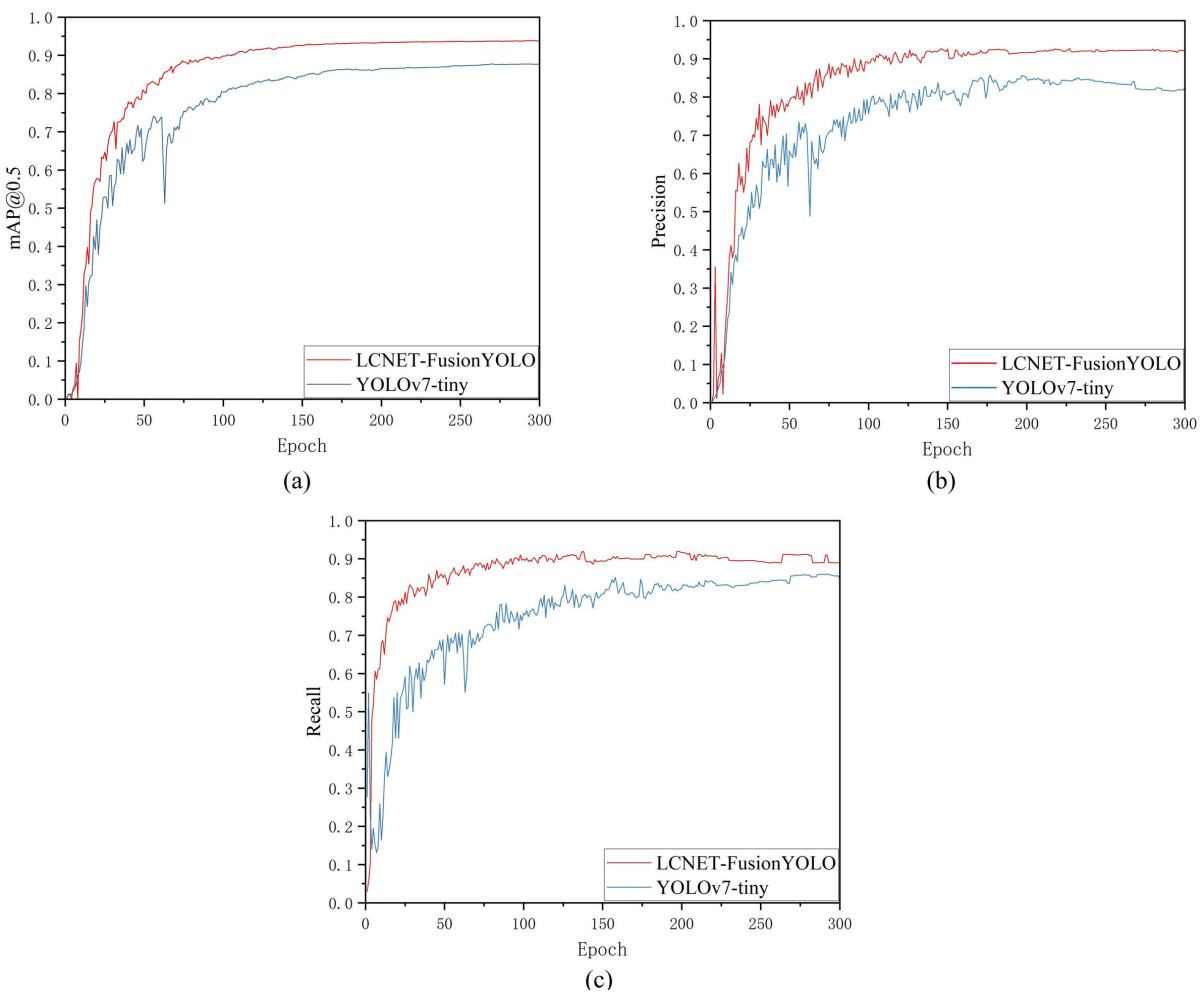
Convergence of indicators: **(a)** The mAP@0.5 variation curve of LCNET-FusionYOLO and YOLOv7-tiny; **(b)** The Precision variation curve of LCNET-FusionYOLO and YOLOv7-tiny; **(c)** The Recall variation curve of LCNET-FusionYOLO and YOLOv7-tiny.

**Figure 12** presents convergence analysis comparing baseline YOLOv7-tiny with LCNET-FusionYOLO across training iterations. The visualization reveals LCNET-FusionYOLO’s superior optimization characteristics: accelerated convergence, enhanced asymptotic performance, and improved training stability across all metrics, with particularly pronounced advantages in mAP@0.5 convergence dynamics. YOLOv7-tiny has an unstable Jaccard index when using the AdaDelta optimizer, and this is caused by five flaws. (a) Rigid pruning leads to poor depth-width coupling, no adaptive modules, unbalanced gradient propagation and high-frequency oscillations. LCNet-FusionYOLO (with PP-LCNet and four core modules) uses ECA, BiECAFusion and DyHead to stabilize the Jaccard index. (b) CIoU loss has sharp curvature, which causes gradient spikes and coordinate shifts. LCNet-FusionYOLO’s Shape-IoU ensures gradual regression. (c) Unidirectional PANet dilutes small-target features. BiECAFusion enables bidirectional feature flow to retain these features. (d) The static detection head is sensitive to semantic-scale drift. DyHead’s real-time reparameterization avoids step changes in the Jaccard index. (e) YOLOv7-tiny’s parameter manifold is non-convex, making AdaDelta oscillate. LCNet-FusionYOLO’s implicit regularization smooths gradient updates, resulting in stable Jaccard index curves.

### 3.2 Comparison of pruning ablation experiments

Post-pruning evaluation employed the speed_up metric, defined as the computational ratio between pruned and unpruned architectures. This metric quantifies efficiency gains, where speed_up = 1.6 corresponds to a 37.5% computational reduction (1 - 1/1.6). The pruning protocol systematically identifies and eliminates redundant structures while preserving essential architectural components. Subsequently, iterative fine-tuning recovers potential performance degradation induced by sparsification.

After LAMP pruning, the model was fine-tuned. It used the same SGD optimizer as pre-pruning training. Key hyperparameters were adjusted to suit the sparse architecture. This ensured stable convergence and performance retention. Core settings are as follows: initial learning rate (lr0) = 0.001, which was reduced from pre-pruning 0.01 to avoid gradient explosion in the sparse model; final learning rate (lrf) = 0.01, which is consistent with pre-pruning and calculated as lr0 × lrf; momentum = 0.937; weight decay = 0.0005; warmup epochs = 3.0; warmup momentum = 0.8; warmup bias lr = 0.1.

Fine-tuning lasted 200 epochs, which is the same as pre-pruning. The learning rate decayed linearly from lr0 to lrf×lr0. We selected lr0 = 0.001 instead of 0.01. The pruned model is more sensitive to high learning rates. Tests showed lr0 = 0.01 caused unstable convergence. lr0 = 0.001 balanced convergence speed and detection performance.

To determine the optimal speed_up, we tested targets from 1.0 to 2.43 with a layer-adaptive magnitude pruning strategy. At speed_up = 2.2, PLFYNet hits 94.5% mAP@0.5 (0.4% higher than pre-pruning LCNET-FusionYOLO). Its parameters drop to 2.98M (50.5% reduction), enabling 50.5 FPS on Jetson Orin Nano (meets <50ms/frame for edge use). Though speed_up = 2.43 is the upper limit, it causes over-pruning: mAP@0.5 falls to 94.2%. Thus, speed_up = 2.2 balances efficiency and performance best. Data supports this in **Table 6**.

**Table 6:** Results of different speed_up data.

Speed_up	Parameter(number)	Memory(MB)	mAP@0.5 (%)
1.0	6.2	10.2	94.1
1.2	5.2	9.3	94.2
1.4	4.4	8.6	94.2
1.6	3.9	7.6	94.3
1.8	3.4	6.7	94.4
2.0	3.1	6.2	94.4
2.2	3.0	5.9	94.5
2.4	2.9	5.7	94.2

### 3.3 Comparison with mainstream object detection models

In this subsection, the final improved model LCNET-FusionYOLO is experimentally compared with eight other common models. The specific experimental results are shown in **Table 7**.

**Table 7:** Results of comparison with mainstream object detection models.

Model	Parameter (million)	Precision (%)	Recall (%)	mAP@0.5 (%)
Faster R-CNN	41.1	83.2	90.1	86.9
SSD	26.3	87.6	88.6	90.3
DEIM-n	3.6	89.3	90.2	90.5
YOLOv3	61.5	87.8	73.7	84.6
YOLOv5s	7.2	91.1	90.2	92.1
YOLOv8n	3.2	92.4	89.7	92.6
YOLOv11n	2.6	92.6	91.2	93.0
RT-DETR-r18	20.1	92.7	89.1	92.9
LCNET-FusionYOLO	6.2	94.1	91.8	94.1
PLFYNet	3.0	94.2	92.1	94.5

The LCNET-FusionYOLO and PLFYNet models were experimentally compared with eight other common object detection models in terms of their mAP@0.5 metrics.

**Figure 13:**
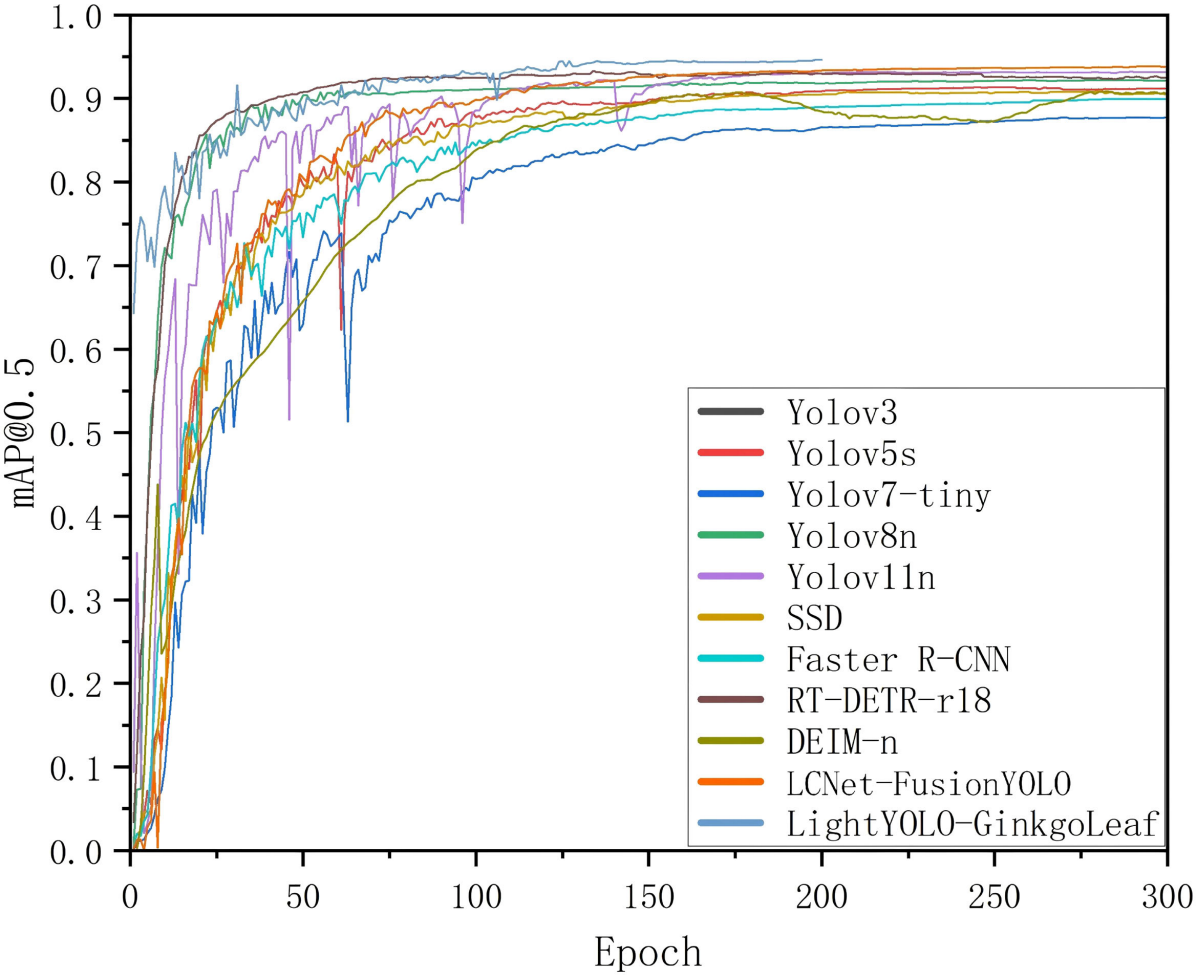
Comparison of mAP0.5 Among LCNET-FusionYOLO, PLFYNet, and eight other common models.

The optimization pipeline employed LAMP pruning for systematic weight elimination, followed by structured sparsification and 200-epoch fine-tuning to mitigate performance degradation. This dual-phase optimization yielded PLFYNet, which demonstrates a 0.4% mAP@0.5 improvement over LCNET-FusionYOLO post-refinement, as illustrated in **Figure 13**.

Comparative analysis against established architectures reveals PLFYNet ‘s superior efficiency-performance trade-off. Two-stage detectors exemplified by Faster R-CNN achieve competitive recall (90.1%) but suffer from computational intractability (137M parameters) and inferior precision (83.2%) relative to single-stage alternatives. SSD’s multi-scale prediction paradigm reduces complexity (26.3M parameters) yet exhibits suboptimal detection metrics (mAP@0.5: 90.3%, recall: 88.6%). DEIM-n’s architectural constraint—utilizing only P4/P5 feature levels—inherently limits small object detection capability, yielding mAP@0.5 of 90.5%. PLFYNet finally surpasses RT-DETR-r18 by 1.5% in Precision, 3% in Recall, and 1.6% in mAP@0.5, demonstrating superior performance.

Within the YOLO lineage, evolutionary progression demonstrates continuous refinement. YOLOv3’s architectural simplicity (61.53M parameters) correlates with inadequate performance (recall: 73.7%, mAP@0.5: 84.6%). Contemporary variants YOLOv5s and YOLOv8n leverage depthwise separable convolutions to achieve substantial compression (7.22M and 3.16M parameters respectively), though performance remains inferior to PLFYNet. Our proposed architecture, with merely 3.0M parameters, achieves state-of-the-art metrics (precision: 94.2%, recall: 92.1%, mAP@0.5: 94.5%)—surpassing Faster R-CNN by 7.6 and SSD by 4.2 percentage points while utilizing 2.2%-21.8% of their parameters. This validates the efficacy of integrated pruning and architectural optimization in achieving unprecedented efficiency without compromising detection fidelity.

**Figure 14:**
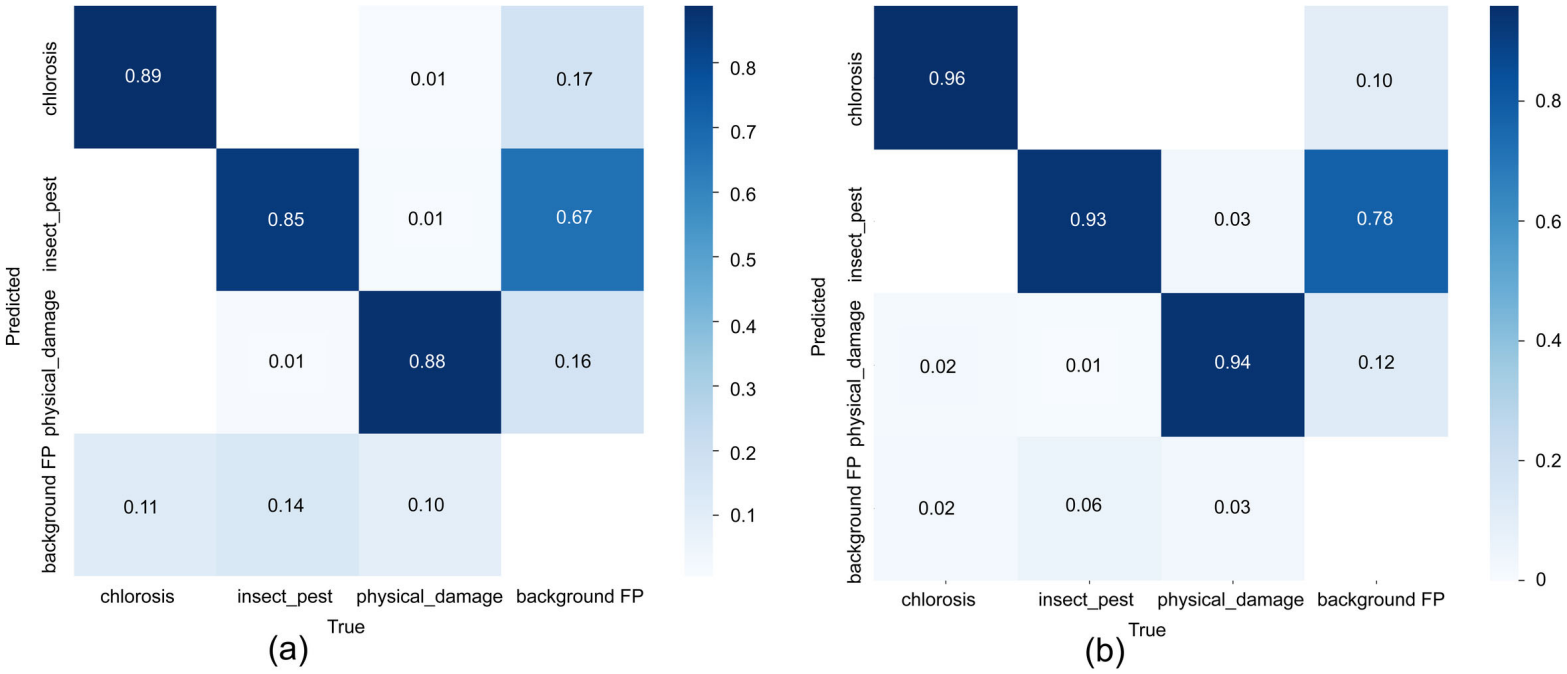
Confusion matrix :**(a)** Confusion matrix of YOLOv7-tiny; **(b)** Confusion matrix of PLFYNet.

The normalized confusion matrices show that YOLOv7-tiny achieves recall rates of 0.89, 0.85, and 0.88 for chlorosis, insect_pest, and physical_damage, respectively, with a background recall of 0.67 and a main false-positive rate of background misclassified as chlorosis (0.17). In contrast, PLFYNet significantly improves the recall of the three disease classes to 0.96, 0.93, and 0.94, markedly reducing missed detections of insect_pest and physical_damage; however, background recall drops to 0.78. When the model training incorporates over-augmented background noise, the chlorosis false-positive rate increases to 0.12. Overall, PLFYNet markedly enhances disease detection accuracy by strengthening foreground feature extraction at the cost of diminished background discriminability, exhibiting a biased improvement of “more accurate foreground, more confused background, “ making it suitable for applications demanding high sensitivity to diseases. It is shown in **Figure 14**.

### 3.4 External validation of the public dataset

**Table 8** compares the core performance metrics of two models in the relevant task (inferred as plant disease detection based on data characteristics). Among them, PLFYNet performs better, with its precision (56.1%), recall (55.9%), and mAP@0.5 (55.7%) all higher than those of YOLOv7-tiny, indicating that the former is superior in recognition accuracy, missed detection control, and comprehensive detection capability. Our Ginkgo-focused dataset has unique traits and high-quality data, but PlantDoc covers 13 plants and 17 diseases with low-quality data. PLFYNet, pruned for edge use, lacks generalization and feature learning, causing low mAP@0.5. PlantDoc’s uneven distribution of different types of labels leads to a significant reduction in learning outcomes. However, the results are still improved compared to the original Yolov7-tiny model, which is more suitable for marginalised deployments.

**Table 8:** External validation results of the public dataset PlantDoc.

Models	Precision (%)	Recall	mAP@0.5
YOLOv7-tiny	51.8%	55.7%	52.4%
PLFYNet	56.1%	55.9%	55.7%

### 3.5 Models deployed on Jetson Orin Nano

#### 3.5.1 Hardware and environment

The Jetson Orin Nano hardware configuration is shown in **Table 9** below:

**Table 9:** Jetson Orin Nano hardware configuration.

Names	Specifications
CPU	6-core Arm^®^ Cortex^®^-A78AE v8.2 64-bit CPU 1.5MB L2 + 4MBL3
GPU	NVIDIA Ampere architecture with 1024 CUDA cores and 32 tensor cores
Memory	8GB 128-bit LPDDR5

Jetson Orin Nano Environment Setup: Install Ubuntu 20.04 operating system, configure the model runtime environment with JetPack 5.1, Python 3.8, PyTorch 1.10.0, TorchVision 0.11.0, CUDA 11.8, and CUDNN 8.6. Integrate a CSI camera for hardware acceleration.

#### 3.5.2 Hardware modules and configurations

The hardware deployment configuration centers on the Jetson Orin Nano platform, integrated with essential peripherals including autonomous power management, visual acquisition system, and wireless communication interface for field operations. Mobile deployment utilizes a ROS-enabled robotic platform manufactured by Helloblock, featuring compact form factor, modular architecture, and versatile hardware integration capabilities for dynamic agricultural monitoring applications.

#### 3.5.3 comparison deployed on Jetson Orin Nano

For on-site power supply, we used a 12V/5A battery. During continuous inference, the model’s average power consumption was 9.8W—17.6% lower than the baseline YOLOv7-tiny model (11.9W). To verify robustness, tests were conducted under extreme backlight, backlighting, or canopy occlusion conditions, with an average inference time of 14.6ms (9.5ms in the laboratory environment), which meets the <50ms threshold required for real-time accurate decision-making.

**Figure 15:**
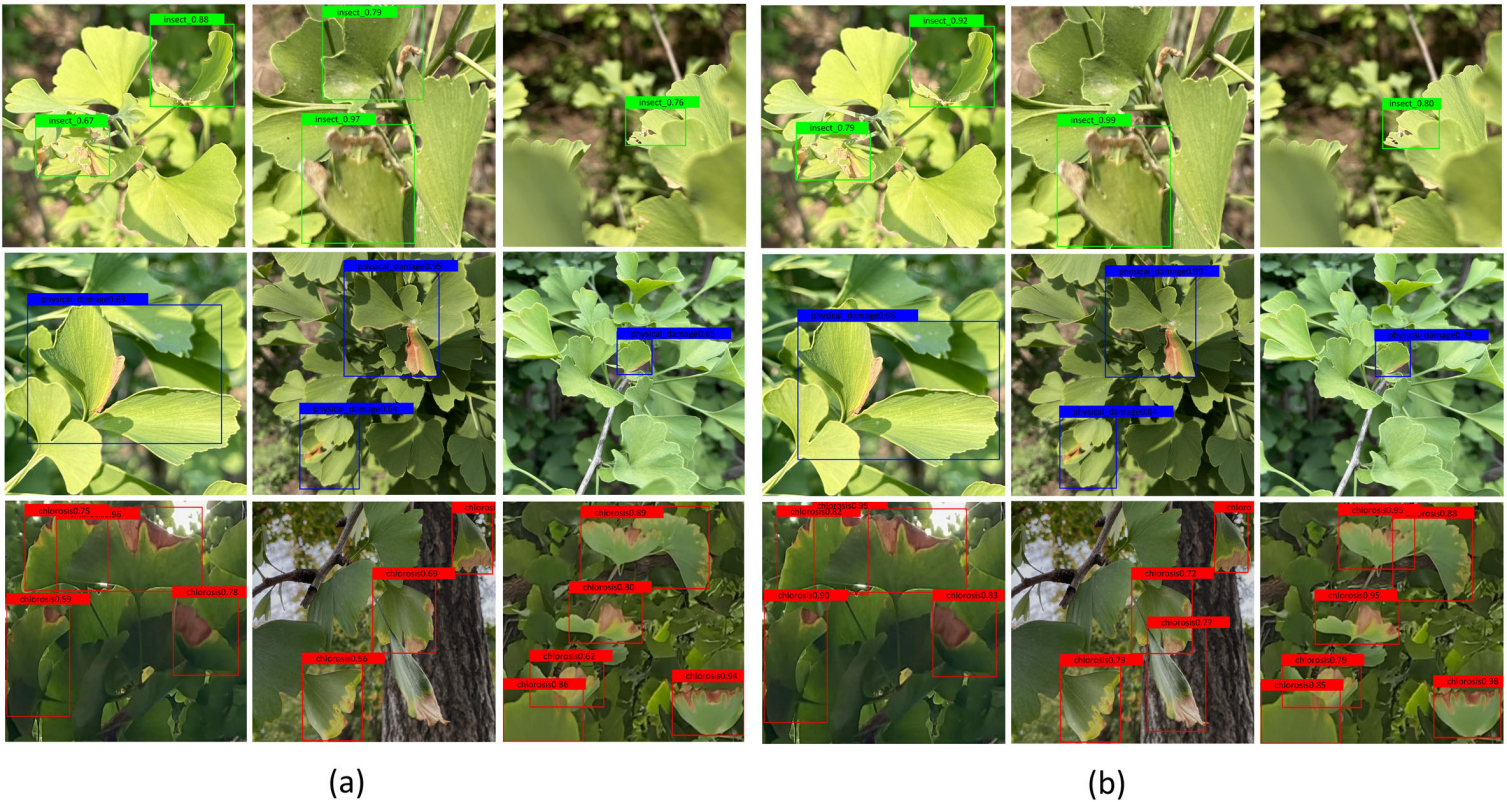
The detection results of the YOLOv7-tiny and PLFYNet on Jetson Orin Nano: **(a)** YOLOv7-tiny; **(b)** PLFYNet.

**Table 10** presents the real-time detection speed comparison of the three models on Jetson Orin Nano. **Figure 15** shows the specific detection results of YOLOv7-tiny (**Figure 15a**) and PLFYNet (**Figure 15b**) after final deployment, with the two subfigures comparing their display effects. They respectively demonstrate the detection performance of the three labels (insect pest, physical damage, and chlorosis), and clearly, PLFYNet achieves better detection accuracy.

**Table 10:** Speed comparison of real-time detection speed of three models on Jetson Orin Nano.

Model	GFLOPS	Inference (ms)	NMS (ms)	FPS
YOLOv7-tiny	13.2	13.1	2.2	41.2
LCNET-FusionYOLO	13.6	13.5	2.6	40.3
PLFYNet	6.2	9.5	1.9	50.5

## 4 Conclusions

Addressing the critical requirements for real-time Ginkgo biloba leaf disease detection in edge computing scenarios, this study presents PLFYNet, a lightweight deep learning model that resolves the deployment challenges of high-precision disease detection systems on resource-constrained embedded devices while maintaining detection accuracy comparable to computationally intensive models.

This research makes three main contributions.First, we systematically evaluated five lightweight backbone architectures: MobileNetV3, GhostNet, ShuffleNetV2, PP-PicoDet, and PP-LCNet. We found that the reconstructed PP-LCNet is the optimal backbone for Ginkgo disease detection. Compared to YOLOv7-tiny, it reduces parameters by 26.8% while maintaining competitive accuracy. Second, we developed BiECAFusion to replace PANet. BiECAFusion includes 1×1 convolutions for channel alignment, ECA attention for dynamic weighting, and bidirectional feature interaction (x_0_ + w_1_x_1_, x_1_ + w_0_x_0_). This addresses the small target feature loss inherent in unidirectional fusion.We combined BiECAFusion with Bottleneck Transformer, Shape-IoU loss, and DyHead to create LCNET-FusionYOLO. This model achieves 94.1% mAP@0.5 with 6.21M parameters.Third, we used Layer-Adaptive Magnitude-based Pruning (LAMP) to further compress the model. The model’s parameters were reduced to 3.0M (a 50.5% reduction), and its mAP@0.5 was improved to 94.5%. This shows that strategic pruning boosts both model efficiency and accuracy, ultimately resulting in the PLFYNet model.

Deployment on Jetson Orin Nano validated practical applicability: the model achieved 50.5 FPS inference speed (22.6% improvement over YOLOv7-tiny’s 41.2 FPS) with 94.2% precision and 92.1% recall across three disease categories (chlorosis, insect pest, physical damage). Comparative analysis against eight mainstream detectors revealed superior mAP@0.5 performance using only 2.2%-21.8% of traditional two-stage detector parameters.

Current limitations include: (1) dataset geographical constraints to Jiangsu Province, China, potentially limiting generalization; (2) unexplored performance under extreme weather and illumination conditions; (3) pruning strategy requiring extensive fine-tuning, suggesting opportunities for more efficient compression methods.

Future work encompasses: This study lays a foundation for practically edge-deployable disease detection in precision agriculture and has broad implications for AI-driven sustainable farming practices. Future research will cover multiple dimensions: in terms of data, expand datasets across diverse geographical and environmental conditions, dynamically adjust augmentation strategies based on natural sample distribution, standardize annotation processes via cross-regional collaboration, introduce an augmentation-validation feedback loop, and verify annotation consistency with Kappa coefficient (target ≥ 0.90); in terms of model & hardware, explore knowledge distillation to reduce complexity without accuracy loss, develop adaptive pruning frameworks for hardware-specific optimization, and integrate multi-spectral imaging to enhance disease characterization; in terms of deployment, develop an offline-first mode by integrating LoRa modules. These measures aim to fully improve technical implementation feasibility.

## Data Availability

The original contributions presented in the study are included in the article/**Supplementary Material**. Further inquiries can be directed to the corresponding author.
