# Supplementary figures and images for: PLFYNet-based edge-deployable detection system for Ginkgo biloba leaf diseases
Source: Front Plant Sci. 2025 Nov 27;16:1679455. doi: 10.3389/fpls.2025.1679455 (PMC12696162; doi:10.3389/fpls.2025.1679455)

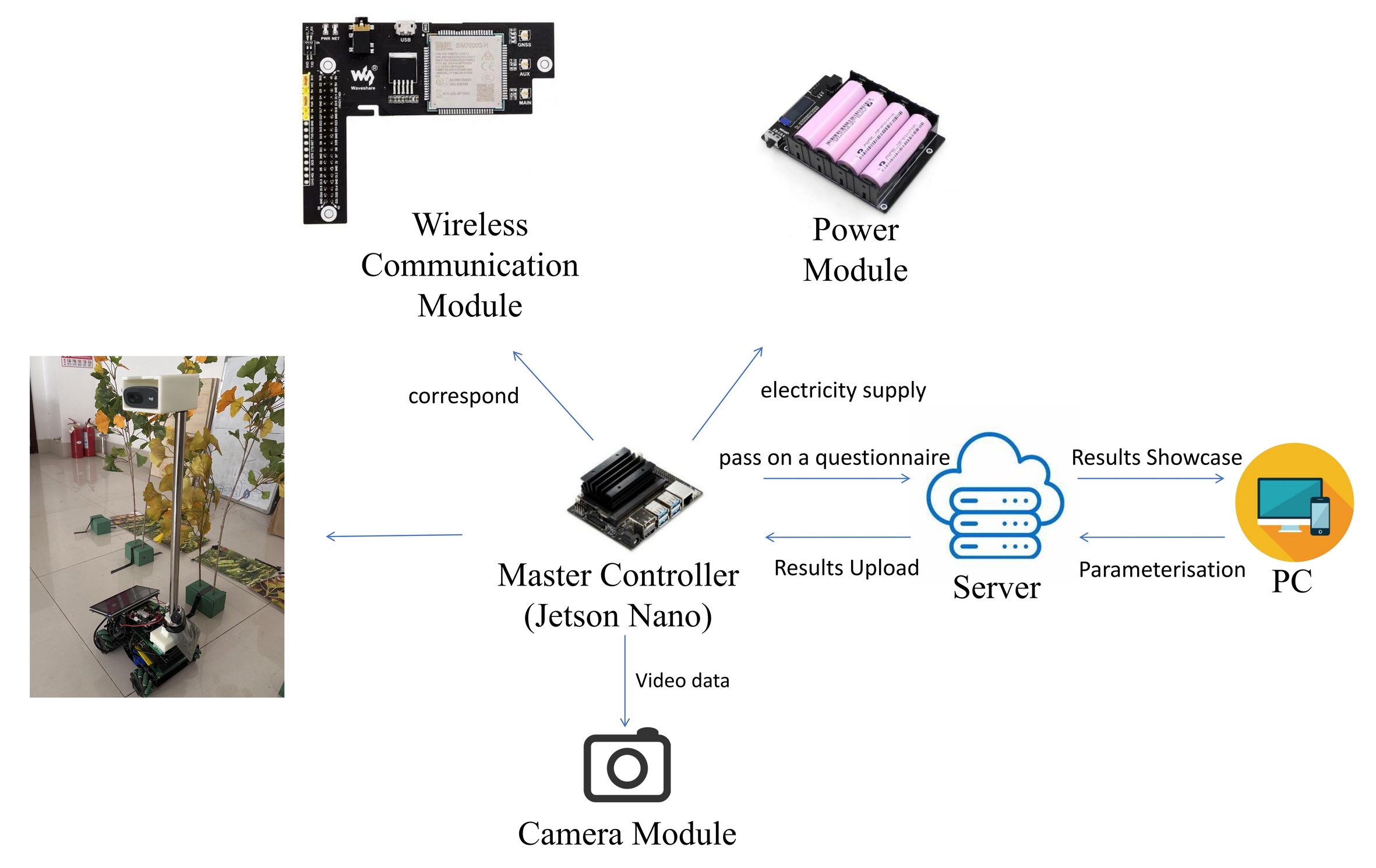

Supplement: Supplementary file 1 [file Image1.jpeg]
