# Supplementary material for: PLFYNet-based edge-deployable detection system for Ginkgo biloba leaf diseases
Source: Front Plant Sci. 2025 Nov 27;16:1679455. doi: 10.3389/fpls.2025.1679455 (PMC12696162; doi:10.3389/fpls.2025.1679455)
Supplement: Supplementary file 2 [file DataSheet1.docx]

Supplementary Material

# Supplementary Data

**Shape-IoU loss function**：
The Shape-IoU loss function is calculated as follows:

| $IoU=\frac{\left\vert B\bigcap B^{gt} \right\vert}{\left\vert B\bigcup B^{gt} \right\vert}$ | (1) |
| --- | --- |
| $ww=\frac{2\times\left( w^{gt} \right)^{scale}}{\left( w^{gt} \right)^{scale}+\left( h^{gt} \right)^{scale}}$ | (2) |
| $hh=\frac{2\times\left( h^{gt} \right)^{scale}}{\left( w^{gt} \right)^{scale}+\left( h^{gt} \right)^{scale}}$ | (3) |
| ${distance}^{shape}=hh\times{\left( x_{c}-x_{c}^{gt} \right)^{2}}/{c^{2}}+ww\times{\left( y_{c}-y_{c}^{gt} \right)^{2}}/{c^{2}}$ | (4) |
| $\Omega^{shape}=\sum_{t=w,h} {(1-e^{-\omega_{t}})}^{\theta},\theta=4$ | (5) |
| $\left\{ \begin{aligned} \omega_{w}=hh\times\frac{\vert w-w^{gt}\vert}{max(w,w^{gt})} \\ \omega_{h}=ww\times\frac{\vert h-h^{gt}\vert}{max(h,h^{gt})} \end{aligned} \right.$ | (6) |

The scale parameter is empirically calibrated based on the object size distribution within the training corpus. Directional weight coefficients, denoted as ww and hh for horizontal and vertical axes respectively, are dynamically computed from the geometric properties of ground truth annotations. The comprehensive bounding box regression objective is formulated as:

| $L_{Shape-IoU}=1-IoU+{distance}^{shape}+0.5\times\Omega^{Shape}$ | (7) |
| --- | --- |

**DyHead：**

DyHead constitutes an innovative detection head architecture . Formally, for a feature tensor F ∈ R^(L×S×C), the self-attention mechanism is expressed as:

| $W\left( \mathcal{F} \right)=\pi\left( \mathcal{F} \right)\mathcal{\cdot F}$ | (8) |
| --- | --- |

In this formulation, π(⋅) represents the attention transformation function. Although fully-connected architectures could theoretically model such high-dimensional interactions, the computational complexity of simultaneously learning across all tensor dimensions renders this approach intractable. Consequently, we adopt a factorized attention strategy, decomposing the operation into three consecutive transformations, with each targeting a specific dimensional axis independently.

| $W\left( \mathcal{F} \right)=\pi_{C}\left( \pi_{S}\left( \pi_{L}\left( \mathcal{F} \right)\mathcal{\cdot F} \right)\mathcal{\cdot F} \right)\mathcal{\cdot F}$ | (9) |
| --- | --- |

$\pi_{L}(\cdot)$, $\pi_{S}(\cdot)$ and $\pi_{C}(\cdot)$ represent dimension-specific attention transformations corresponding to level, spatial, and channel axes, respectively. Sequential execution of these operators ensures computational tractability while preserving inter-dimensional dependencies. The formulation in Equation (9) inherently supports recursive composition, facilitating the construction of deep architectures through cascaded $\pi_{L}$, $\pi_{S}$, and $\pi_{C}$ modules.

The methodology fundamentally balances sparsity optimization with performance preservation through adaptive global pruning. Weight salience is determined via magnitude-based scoring coupled with ℓ₂ distortion minimization at the network level. The algorithmic pipeline comprises:

| $\left[ \begin{aligned} \begin{aligned} u_{1} \\ u_{2} \\ \ldots\end{aligned} \\ u_{n-1} \\ u_{n} \end{aligned} \right]\left[ \begin{aligned} v_{1} \\ v_{2} \\ \ldots\\ v_{n-1} \\ v_{n} \end{aligned} \right] \to\left[ \begin{aligned} \frac{u_{1}^{2}}{u_{1}^{2}} \\ \frac{u_{2}^{2}}{u_{1}^{2}+u_{2}^{2}} \\ \ldots\\ \frac{u_{n-1}^{2}}{u_{1}^{2}+\ldots+u_{n-1}^{n-2}} \\ \frac{u_{n}^{2}}{u_{1}^{2}+\ldots+u_{n}^{n-1}} \end{aligned} \right]\left[ \begin{aligned} \begin{aligned} \frac{v_{1}^{2}}{v_{1}^{2}} \\ \frac{v_{2}^{2}}{v_{1}^{2}+v_{2}^{2}} \\ \ldots\end{aligned} \\ \frac{v_{n-1}^{2}}{\begin{aligned} v_{1}^{2}+\ldots+v_{n-1}^{n-2} \\ \frac{v_{n}^{2}}{v_{1}^{2}+\ldots+v_{n}^{n-1}} \end{aligned}} \end{aligned} \right] \to\left[ \begin{matrix} \frac{u_{1}^{2}}{u_{1}^{2}} & \frac{v_{1}^{2}}{v_{1}^{2}} \\ \frac{u_{1}^{2}}{u_{1}^{2}+u_{2}^{2}} & ф \\ ф & ф \\ ... & ... \end{matrix} \right] (10)$ | | |
| --- | --- | --- |
| sorting according to the magnitude of weights | LAMP Score Calculation | Global pooling and pruning |

**Sorting according to the magnitude of weights:** Parameters within each network layer are arranged in descending order based on their absolute values, establishing a magnitude-based hierarchy. **LAMP Score Calculation:** The algorithm computes normalized importance metrics by evaluating the squared magnitude of each weight relative to the layer's weight distribution, yielding calibrated significance scores. **Global Pooling and Pruning:** Layer-specific scores undergo aggregation into a unified importance repository, followed by comprehensive ranking and systematic parameter elimination based on global thresholds. This methodology achieves optimal compression while preserving essential modelcapabilities.

# 2 Supplementary Figures and Tables

## 2.1 Supplementary Tables

Table 1 below shows some of the relevant hardware deployment parameters.

Table 2 Hardware parameter information

| **Hardware Item** | **Model / Specification** |
| --- | --- |
| Chassis | Medium articulated swing-arm chassis |
| Motor | 520 motor × 4 |
| LiDAR | SLAMTEC A1 |
| Camera | Astra Pro Plus depth camera |
| Display | 8-inch touch monitor |
| Controller | Jetson Orin Nano 8GB |
| Motor Battery | 6000 mAh Li-ion battery pack |
| Controller Battery & Booster | 12 V 5100 mAh battery + 12 V to 19 V boost module |
| Wireless Communication | SIM7600G-H 4G extension board |

## Supplementary Figures

To enable users to detect and observe leaf-used ginkgo diseases in real time and display the detection video stream processed on the Jetson Nano development board on the user side, we have designed a PC-based real-time detection system based on PyQt5. We analyzed user requirements, and the system needs to meet the following functions:

(1) It can accurately detect leaf-used ginkgo diseases;

(2) It allows users to select detection models;

(3) Users can select existing images and transmit them to the development board for detection;

(4) Users can select existing videos and transmit them to the development board for detection;

(5) Users can turn on the camera for real-time detection;

(6) It can clearly display data such as disease types, confidence levels, and disease quantities on the system interface. The specific flow chart is shown in Figure 1 below:

| 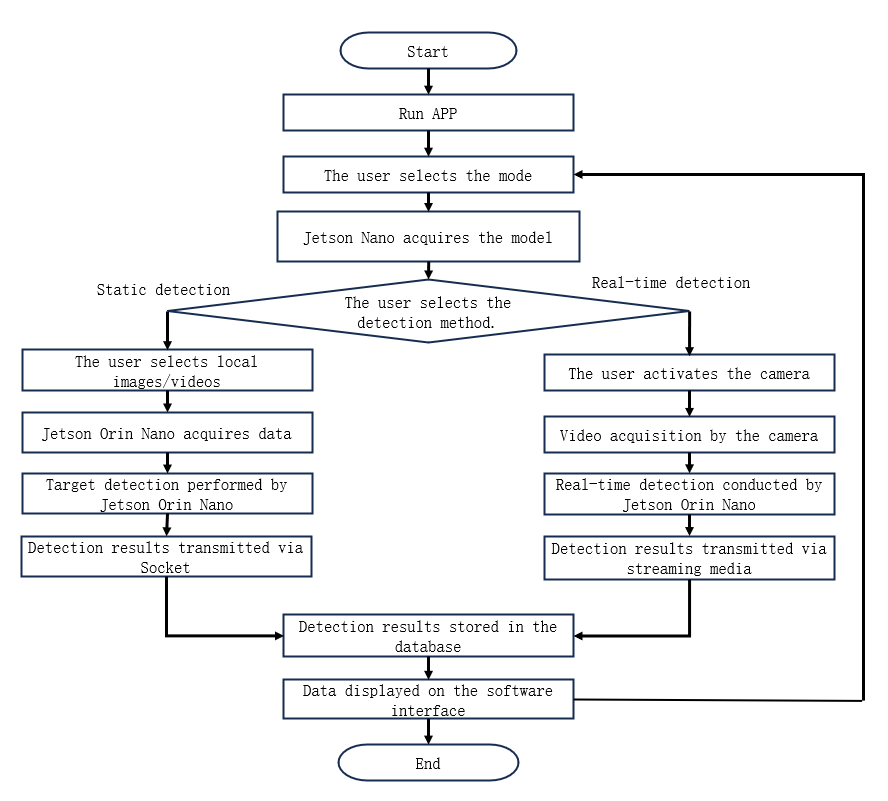 |
| --- |
| Figure 1: Design Flow Chart for Leaf-Used Ginkgo Disease Detection |

Figure 6: QT Detection Interface

| 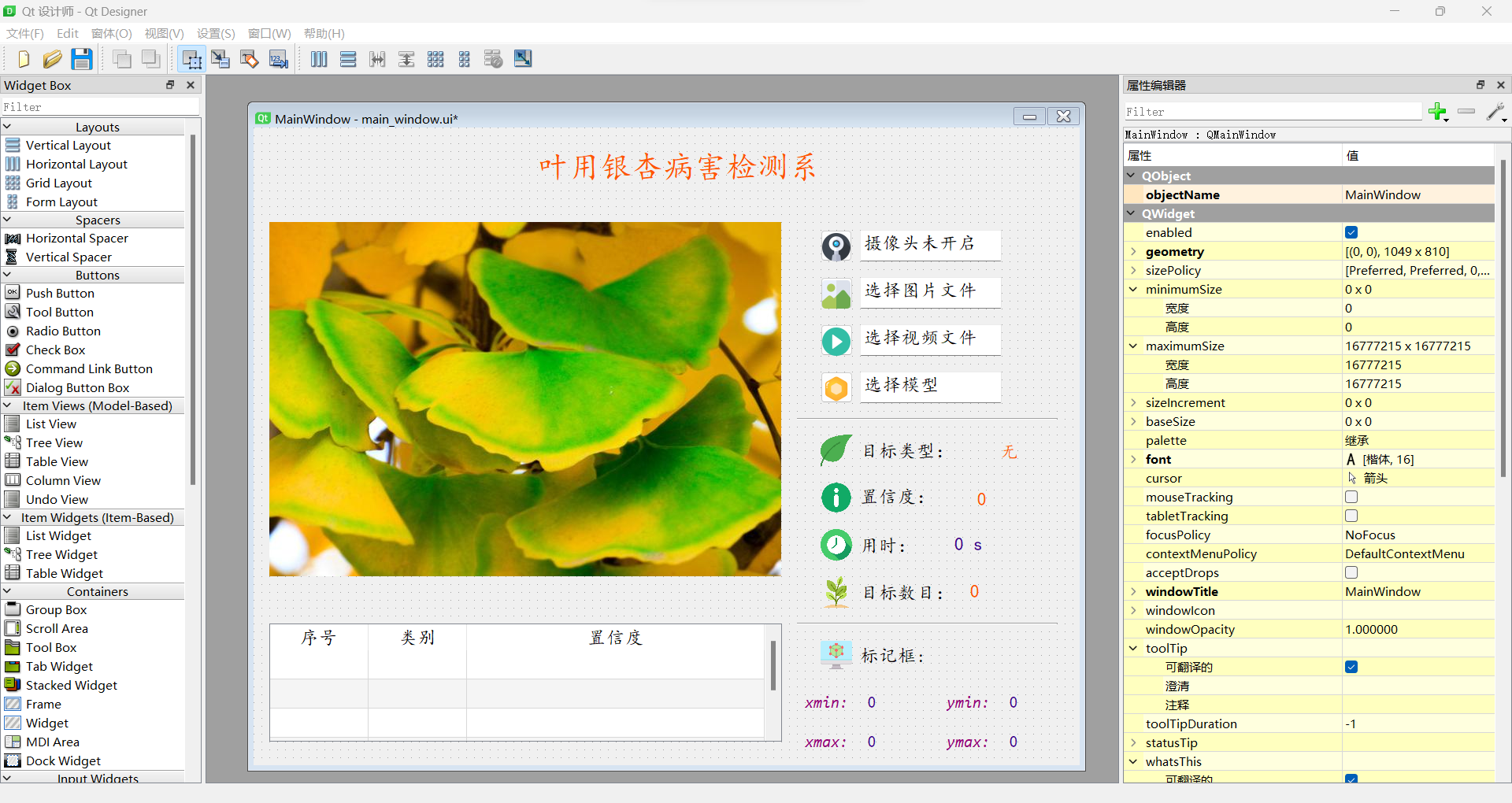 |
| --- |
| Figure 2 QT Detection Interface |

This system adopts a lightweight and high-precision disease detection algorithm, which can accurately identify leaf diseases. It can also upload the detection results and data from the development board to the user-side application in real time through remote communication for display. Moreover, the system is designed with a rich functional interactive interface to meet different disease detection needs. Experimental verification shows that the lightweight remote disease detection system for leaf-used ginkgo developed in this paper can perform real-time disease detection after turning on the camera to collect video information in actual complex scenarios, and can realize low-latency transmission to the user side for display, achieving disease detection of leaf-used ginkgo in practical scenarios.

Ginkgo trees have an average height of about 2 meters and are arranged in rows. Their branches and leaves shade each other between the rows, and diseases generally appear in the middle of the trees. However, UAVs cannot penetrate into the rows for detection and are prone to crashing. Therefore, using UAVs for detection in large-scale ginkgo plantations is not feasible, as shown in Figure 3 for the actual growth status of ginkgo plantations.

| 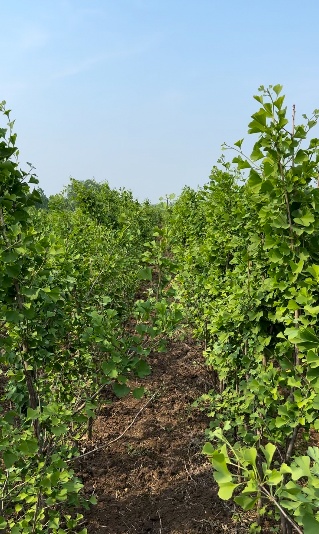 |
| --- |
| Figure 3 Actual Growth Status of Ginkgo Plantations |
